# Deficiency of *heme oxygenase 1a* causes detrimental effects on cardiac function

**DOI:** 10.1111/jcmm.18243

**Published:** 2024-03-20

**Authors:** Hong Wang, Juuso Siren, Sanni Perttunen, Katariina Immonen, Yu‐Chia Chen, Suneeta Narumanchi, Riikka Kosonen, Jere Paavola, Mika Laine, Ilkka Tikkanen, Päivi Lakkisto

**Affiliations:** ^1^ Minerva Foundation Institute for Medical Research Helsinki Finland; ^2^ Department of Anatomy University of Helsinki Helsinki Finland; ^3^ Heart and Lung Centre University of Helsinki and Helsinki University Hospital Helsinki Finland; ^4^ Abdominal Centre Nephrology University of Helsinki and Helsinki University Hospital Helsinki Finland; ^5^ Department of Clinical Chemistry University of Helsinki and Helsinki University Hospital Helsinki Finland

**Keywords:** cardiac hypertrophy, cardiac output, heme oxygenase 1, interstitial fibrosis

## Abstract

Humans lacking heme oxygenase 1 (HMOX1) display growth retardation, haemolytic anaemia, and vulnerability to stress; however, cardiac function remains unclear. We aimed to explore the cardiac function of zebrafish lacking *hmox1a* at baseline and in response to stress. We generated zebrafish *hmox1a* mutants using CRISPR/Cas9 genome editing technology. Deletion of *hmox1a* increases cardiac output and further induces hypertrophy in adults. Adults lacking *hmox1a* develop myocardial interstitial fibrosis, restrain cardiomyocyte proliferation and downregulate renal haemoglobin and cardiac antioxidative genes. Larvae lacking *hmox1a* fail to respond to hypoxia, whereas adults are insensitive to isoproterenol stimulation in the heart, suggesting that *hmox1a* is necessary for cardiac response to stress. Haplodeficiency of *hmox1a* stimulates non‐mitochondrial respiration and cardiac cell proliferation, increases cardiac output in larvae in response to hypoxia, and deteriorates cardiac function and structure in adults upon isoproterenol treatment. Intriguingly, haplodeficiency of *hmox1a* upregulates cardiac *hmox1a* and *hmox1b* in response to isoproterenol. Collectively, deletion of *hmox1a* results in cardiac remodelling and abrogates cardiac response to hypoxia and isoproterenol. Haplodeficiency of *hmox1a* aggravates cardiac response to the stress, which could be associated with the upregulation of *hmox1a* and *hmox1b*. Our data suggests that HMOX1 homeostasis is essential for maintaining cardiac function and promoting cardioprotective effects.

## 1 INTRODUCTION

Heme oxygenase 1 (HMOX1), a stress‐inducible protein, degrades heme into bioactive signalling molecules carbon monoxide and biliverdin while simultaneously releasing iron.^1^ Human HMOX1 deficiency is a rare autosomal recessive disorder with hallmark features of haemolytic anaemia, inflammation of multiple organs and vasculature, iron deposition, interstitial fibrosis and growth retardation.^2^, ^3^, ^4^, ^5^ An autopsy of a 6‐year‐old boy lacking HMOX1 revealed left ventricular hypertrophy.^6^ In addition, a long GTn polymorphism in the promoter of *HMOX1*, exhibiting low transcriptional activity, is associated with cardiovascular diseases.^7^ Consistent with HMOX1‐deficient patients, HMOX1‐deficient rodents displayed reduced body weight, anaemia, chronic inflammation and dysfunction of multiple organs.^8^, ^9^, ^10^ Thus, the lack of HMOX1 appears to cause detrimental effects on mammalian tissues.

During the past two decades, the cardioprotective effect of HMOX1 induction has been extensively explored.^11^, ^12^, ^13^ Overexpression of HMOX1 before ischemia/reperfusion (I/R) reduces cardiac ischaemic damage, whereas inhibition of HMOX1 activity deteriorates I/R injury.^14^, ^15^ In addition, mice with global and cardiac‐specific overexpression or activation of HMOX1 exhibit markedly lower infarct size and improved cardiac function after cardiac injury.^16^, ^17^, ^18^ In contrast, the lack of HMOX1 in mice results in increased myocardial damage after I/R.^19^ Cardioprotective effects of HMOX1 are mediated by diverse mechanisms, including reduction in oxidative stress, inhibition of apoptosis and modulation of mitochondrial function.^20^, ^21^

Although the cardioprotective effects of HMOX1 exist under diverse stress conditions, induction of HMOX1 has been associated with the development of chronic viral myocarditis.^22^ Systemic overexpression of HMOX1 aggravates cardiac hypertrophy induced by pressure overload and fails to prevent arterial dysfunction upon injury in mice.^23^, ^24^ Despite the association of HMOX1 with protection against oxidative stress,^25^ excess HMOX1 expression contributes to apoptosis mediated by oxidative stress.^22^ HMOX1 appears to mediate pathological crosstalk between macrophages and cardiomyocytes, resulting in increased oxidative stress and cardiomyocyte apoptosis, consequently contributing to heart failure.^22^ Thus, further studies are needed to elucidate the exact role of HMOX1 in various stressful events.

Zebrafish have emerged as a compelling vertebrate model for defining the conserved genetic and molecular basis of cardiac disease and for accessing the therapeutic potential of small molecules. Because of genome duplication events in teleosts, zebrafish have two paralogs of *hmox1*, *hmox1a* and *hmox1b*, showing significant conservation to human *HMOX1*.^26^ Paralogs of *hmox2*, *hmox2a* and *hmox2b*, are closely related to human *HMOX2* homologues.^27^ Similar to human *HMOX1*, zebrafish *hmox1a* and *hmox1b*, but not *hmox2a* and *hmox2b*, display transcriptional responses to additional pro‐oxidant exposure.^26^, ^27^ Previous studies demonstrated a conserved role for *hmox1a*, but not *hmox1b*, in normal macrophage migration to the wound site and in protecting the host from *M. marinum* infection by generating a *hmox1a* mutant via TALENs.^28^, ^29^ In this study, we generated zebrafish *hmox1a* null mutants using CRISPR/Cas9 genome editing to explore the cardiac functional role of *hmox1a* at baseline and in response to stress.

## 2 MATERIALS AND METHODS

The detailed methods are provided in the Supplementary materials.

### 2.1 Generation of zebrafish *hmox1a* mutants

Zebrafish of the Turku line have been maintained in the zebrafish core facility at the University of Helsinki. Animal experiments were approved by the Regional Government Office of Southern Finland in agreement with the ethical guidelines of the European Union (ESAVI/4131/04.10.07/2017; ESAVI/16286/2020). The CRISPR/Cas9‐targeted mutagenesis was performed to generate zebrafish *hmox1a* (NM_001127516.1) mutants. The target site, 5′‐GGAGGCTCTGGGGCAGGACTTGG‐3′, was selected using ZiFiT Targeter software (http://zifit.partners.org/) without predicted off‐target site. Single‐guide RNA (sgRNA) was synthesised using the MAXIscript T7 kit (Life Technologies, Carlsbad, CA, USA). The sequence of *hmox1a* gRNA crRNA:tracrRNA was 5′‐gcgTAATACGACTCACTATAGGAGGCTCTGGGGCAGGACTGTTTTAGAGCTAGAAATAGC‐3′. Cas9 mRNA was synthesized with the plasmid pMLM3613 encoding Cas9 nuclease (Addgene, plasmid #42251) and the mMESSAGE T7 ULTRA kit (Life Technologies). Approximately 200 embryos were co‐injected with Cas9 mRNA and gRNA at the one‐cell stage. Mutated alleles were identified by high‐resolution melting (HRM) analysis (Roche Diagnostics GmbH, Mannheim, Germany) and Sanger sequencing. Ten founders (F0) carrying a 52‐bp deletion in exon 3 of *hmox1a* were outcrossed with the wild type to obtain F1 zebrafish heterozygous mutant. They were subsequently inbred to obtain F2 wild‐type (WT) (*hmox1a* ^+/+^), heterozygous (HET) (*hmox1a* ^+/−^) and homozygous (KO) (*hmox1a* ^−/−^) zebrafish. HET mutants of F2 or later generations were inbred to generate WT, HET and KO embryos used in this study.

### 2.2 Genotyping

We genotyped either adult zebrafish or 3 dpf embryos at each generation as described previously^30^ using the corresponding primers (Table S1). Each genotype displayed a distinct melting curve.

### 2.3 Cardiac function of zebrafish larvae

The cardiac function of larvae of mixed gender at 5–6 dpf was analysed as described earlier.^31^ Ventricular volume was calculated based on ventricular width and length at the ends of diastole and systole. Ejection fraction (EF), stroke volume (SV) and cardiac output (CO) were calculated on the basis of ventricular volume. Five diastoles and systoles were analysed for each heart.

### 2.4 Echocardiography of adult zebrafish

Echocardiography of adult zebrafish of mixed gender was performed with a Vevo 2100® Image System (VisualSonics, Amsterdam, Netherlands) equipped with a high‐frequency transducer (MS700, 30–70 MHz) as described previously.^32^ B‐mode videos were recorded in the longitudinal axis view, and pulsed‐wave Doppler (PWD) signals were obtained in the short axis view. EF, SV and CO were determined from B‐mode images. The maximal velocity of blood inflow across the atrioventricular valve during early diastole (E wave), atrial systole (A wave), and deceleration time of E wave were derived from PWD signals. HR was also obtained from the PWD image.

### 2.5 Hypoxia exposure in zebrafish larvae

At 4 dpf, larvae were exposed to hypoxia (3% O_2_) at 28°C in a Heracell VIOS 160i incubator (Thermo Fisher Scientific Inc., Waltham, MA, USA). Nitrogen gas was bubbled into the sealed incubator to maintain the oxygen conditions. Oxygen concentrations were measured with a Fibox 3 fibre optic oxygen probe and transmitter (PreSens Precision Sensing GmbH, Regensburg, Germany). Half of the embryos from each genotype were placed under hypoxic conditions, and half were placed under normoxic condition. After 24‐h exposure, larvae were immediately subjected to either cardiac function analysis or snap‐frozen.

### 2.6 Zn(II) Protoporphyrin IX treatment in adult zebrafish

Wild‐type adult zebrafish were anaesthetized with 0.02% tricaine in system water and i.p. injected with Zn(II) protoporphyrin IX (ZnPPIX, Frontier Scientific, Logan, UT, USA) at a dose of 5 mg/kg body weight on days 1 and 7. Control zebrafish received saline. Echocardiographic examinations were performed before and at 13 dpi.

### 2.7 Isoproterenol treatment in larval and adult zebrafish

Zebrafish embryos received 300 μM isoproterenol (ISO; Sigma‐Aldrich, Munich, Germany) at 2 dpf for 4 days. Larval cardiac function was monitored at 6 dpf. Adult zebrafish of mixed gender were anaesthetized with 0.02% tricaine in system water and i.p. injected with a single high dose of ISO at 150 mg/kg body weight.^33^ Fish from each genotype were randomly divided into two groups based on body size: control and ISO. Control zebrafish received saline.

### 2.8 Primary cardiomyocyte isolation

Primary cardiomyocytes (CMs) were isolated and purified from adult ventricles of KO(52del), HET(52del) and WT zebrafish, as previously described.^34^ Purified cardiomyocytes were seeded in poly l‐lysine‐coated cell culture plates (Agilent Seahorse XF96 Cell Culture Microplates, Santa Clara, CA, USA) at 10,000 cells/well and cultured at 28°C in 5% CO_2_.

### 2.9 Cardiomyocyte energy metabolism

The oxygen consumption rate (OCR) and extracellular acidification rate (ECAR) in 4‐day primary CMs were measured with an XF Mito Stress Test Kit (Agilent) using a Seahorse XF^e^96 analyzer (Agilent) following the manufacturer's instructions. Prior to the assay, the culture medium was replaced with XF assay medium DMEM supplemented with 10 mM glucose, 2 mM glutamine and 1 mM pyruvate. During the assay, basal OCR and ECAR were measured, followed by sequential injections of 2 μM oligomycin, 3 μM carbonyl cyanide‐*p*‐trifluoromethoxyphenylhydrazone (FCCP), and 1 μM rotenone/antimycin A through drug injection ports. During the last injection step, cells were simultaneously stained with 2 μM Hoechst 33342 (Thermo Scientific, Rockford, IL, USA) to determine cell number using the Cytation 5 Cell Imaging Multi‐Mode Reader (Biotek, Agilent Technologies). For the ISO treatment experiment, cardiomyocytes isolated from HET(52del) and wild‐type adult hearts were treated with 10 μM ISO for 24 h prior to Seahorse assay. Data were analysed with Seahorse Wave software.

### 2.10 Western blotting

Pooled zebrafish larvae were lysed in chilled RIPA buffer supplemented with phosphatase and protease inhibitors (Roche) using a sonicator (Sonopuls HD2070, Bandelin, Berlin, Germany). Western blotting was performed as previously described.^35^ The antibodies used are listed in Table S2.

### 2.11 RNA extraction and real‐time quantitative RT‐PCR

RNA extraction was performed with the miRNeasy Mini Kit (Qiagen, Hilden, Germany) according to the manufacturer's instructions. Quantitative RT‐PCR was performed as described^35^ at least three times with three technical replicates for each sample. The primer pairs used are listed in Table S1.

### 2.12 Histology

Acid fuchsin orange G (AFOG) staining (Sigma‐Aldrich, Saint Louis, MO, USA) was performed to detect fibrotic tissue according to the manufacturer's instructions. Slides were digitally scanned using a 3DHISTECH Pannoramic 250 FLASH II (3DHISTECH Ltd., Budapest, Hungary) and quantified with HistoQuant module (3DHISTECH Ltd.).

### 2.13 Immunohistochemistry

Paraffin sections from adult hearts were blocked and stained with antibodies against proliferating cell nuclear antigen (Pcna) and myocyte‐specific enhancer factor 2 (Mef2), followed by incubation with Alexa Fluor‐488‐ and Alexa Fluor‐594‐conjugated secondary antibodies. (Table S2). Nuclei were labelled with DAPI (4′,6‐Diamidino‐2‐Phenylindole, Dihydrochloride) (Molecular Probes, Eugene, OR, USA). Sections were mounted with ProLong Diamond antifade mountant (Molecular Probes) and imaged with a Zeiss LSM 780 confocal microscope (Carl Zeiss Microscopy GmbH, Germany). Quantitative identification of Collagen type I was performed using anti‐mouse Col1A1 antibody (Table S2) and EnVision™^+^ System‐HRP (Dako, Carpinteria, CA, USA) according to the manufacturer's instructions. Slides were digitally scanned using the 3DHISTECH Pannoramic 250 FLASH II and quantified with HistoQuant module. Two sections from each heart were stained, and the ventricular areas from each section were selected for quantification of Collagen type I relative to myocardium‐positive areas.

### 2.14 Statistical analysis

Data were analysed using Prism software (version 10.0; GraphPad, San Diego, CA, USA) and are presented as mean ± SD. One‐way ANOVA with Tukey's adjustment for multiple comparisons was used to calculate differences between more than two groups with normally distributed data. A two‐sample, unpaired, two‐tailed *t*‐test was performed to compare two groups with normally distributed data. Data that did not pass normality and lognormality tests were analysed with the Mann–Whitney test to compare the difference between two groups and with Kruskal‐Wallis followed by Dunn's test in the case of three groups. Statistical significance was set at *p* value <0.05.

## 3 RESULTS

### 3.1 Characterization of the CRISPR/Cas9‐generated zebrafish *hmox1a* mutant allele

**FIGURE 1:**
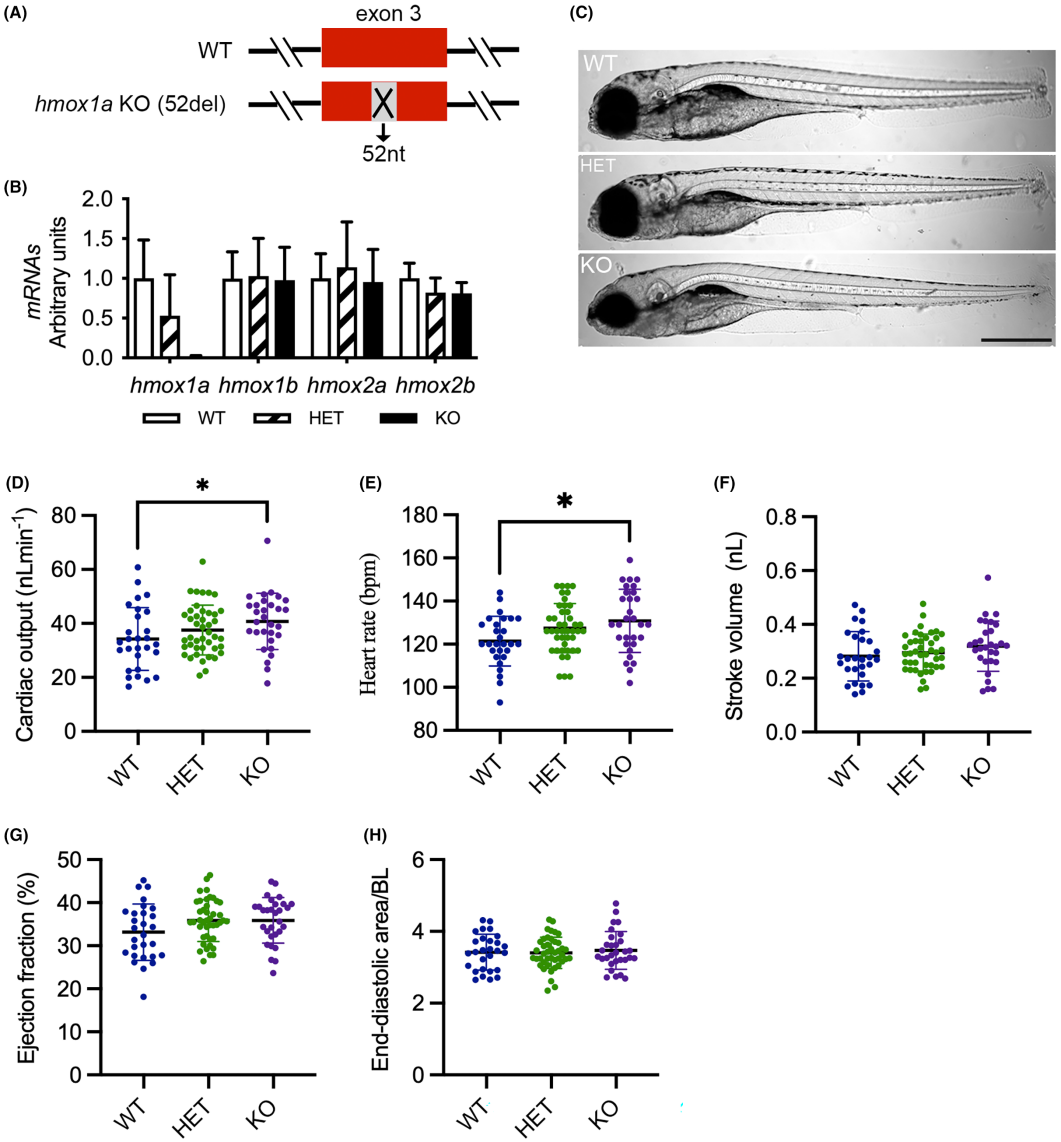
Deletion of *hmox1a* increases cardiac output in larvae. (A) Scheme of CRISPR/Cas9‐generated 52‐base pair deletion in exon 3 of *hmox1a*. (B) RT‐qPCR analysis of *hmox1a*, *hmox1b*, *hmox2a* and *hmox2b* in wild type (WT), heterozygous (HET) and homozygous (KO) *hmox1a* mutant at 5–6 dpf. The results are presented as fold change compared to WT. RNA was extracted from 5 to 10 pooled larvae. The number of RNA samples used for RT‐qPCR analyses as following: WT *n* = 5; HET(52del) *n* = 6; KO(52del) *n* = 6. (C) Representative images of *hmox1a* WT, HET(52del) and KO(52del) larvae at 5 dpf showing no obvious gross phenotype. Scale bar: 500 μm. (D–H) Analysis of cardiac function at 5–6 dpf larvae indicating an increase in cardiac output (D) and heart rate (E), and no significant change in stroke volume (F), ejection fraction (G) and end‐diastolic ventricular area normalized to body length (H) in *hmox1a* KO(52del). WT *n* = 28; HET(52del) *n* = 43; KO(52del) *n* = 30. Data are presented as mean ± SD. One‐way ANOVA with Tukey adjustment for multiple comparisons. *p < 0.05.

We generated a zebrafish *hmox1a* mutant bearing a 52‐bp deletion in exon 3, hereinafter assigned as *hmox1a* (52del) (Figure 1A). The lesion resulted in a frameshift and a premature termination codon (Figure S1). We further investigated the expression of *hmox1a* in wild‐type (WT, *hmox1a* ^+/+^), heterozygous (HET, *hmox1a* ^+/−^), and knockout (KO, *hmox1a* ^−/−^) offspring from F2 HET inbreeding by qRT‐PCR with a primer set spanning the exon 3 and 4 regions. As expected, the expression of *hmox1a* was almost undetectable in the KO(52del) larvae and markedly downregulated by approximately 50% in HET(52del) compared to WT (Figure 1B). To verify the possible genetic compensation response triggered by the deficiency of *hmox1a*, we quantitatively analysed the expression of the paralog *hmox1b* and the isoforms *hmox2a* and *hmox2b*. None of these related genes showed alteration at the transcriptional level in KO(52del) and HET(52del) compared to WT (Figure 1B), indicating that lack of *hmox1a* did not trigger a genetic compensation response. However, investigation of the protein level of Hmox1a was halted by the lack of an antibody specific to zebrafish. Nevertheless, the offspring of HET mutants followed Mendelian inheritance patterns. Both heterozygous and homozygous embryos displayed no gross morphological abnormalities (Figure 1C). They were adult viable and swam normally, but displayed a reduction in spawning frequency.

### 3.2 Deletion of *hmox1a* elevates cardiac output in larvae

We next evaluated the impact of *hmox1a* deficiency on cardiac function in zebrafish larvae. At 5–6 dpf, KO(52del) larvae displayed increased CO (*p* = 0.04) and HR (*p* = 0.03) in comparison with WT, whereas SV and EF showed no significant alteration in KO(52del) larvae (Figure 1D–G). EDA normalised to body length (BL) showed no obvious difference between the mutants and WT larvae (Figure 1H). Taken together, the deletion of *hmox1a* increases CO, which is ascribed to the enhanced HR in zebrafish larvae.

### 3.3 Deletion of *hmox1a* induces cardiac remodelling in adults

**FIGURE 2:**
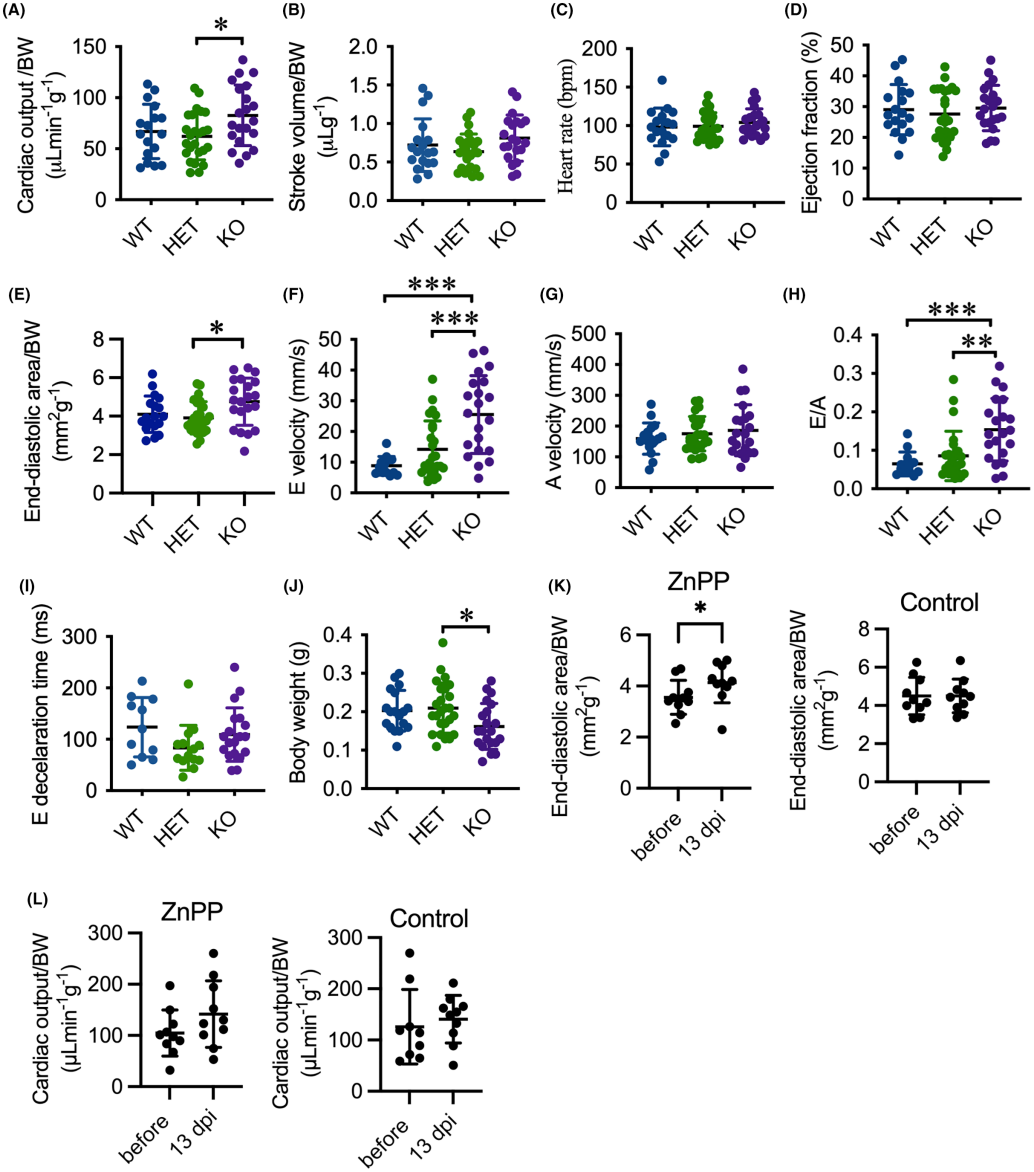
Deletion of *hmox1a* provokes cardiac output and hypertrophy in adults. (A–D) Echocardiography analysis depicting elevated cardiac output in KO(52del) zebrafish compared to their HET sibling (A) with a tendency toward increase in stroke volume (B) and no significant change in heart rate (C) and ejection fraction (D). (E) KO(52del) zebrafish exhibits enlarged end‐diastolic area compared to HET siblings. (F–H) PWD analysis indicating increased peak E wave velocity (F) and E/A ratio (H) without significant change on Peak A wave velocity (G). (I) Deceleration times of E wave from maximum velocity to baseline display no significant difference between the three genotypic groups. (J) KO(52del) zebrafish are lean in comparison with their HET siblings. (K) ZnPPIX treatment leads to enlarged end‐diastolic area in WT zebrafish at 13 dpi. Zebrafish treated with saline display no change at 13 dpi. (L) ZnPPIX treatment induces a tendency toward increase in cardiac output in WT zebrafish at 13 dpi. Zebrafish treated with saline display no change at 13 dpi. (A–J) WT *n* = 18; HET(52del) *n* = 25; KO(52del) *n* = 21. K, L ZnPPIX *n* = 10; Control *n* = 10. Data are presented as mean ± SD. One‐way ANOVA with Tukey adjustment for multiple comparisons in A–J. Two‐sample *t*‐test in K, L. *p < 0.05, **p < 0.01, ***p < 0.001.

We further investigated the cardiac function of adult *hmox1a* mutants at 6 months of age by echocardiography. A previous study indicated that normalising for body weight facilitates standardised comparison of derived ventricular volume between groups of zebrafish of different age and gender.^32^ Accordingly, we normalised ventricular area and volume to body weight. KO(52del) zebrafish showed an increase in CO by 26.3% and in SV by 12.4% compared to WT, but neither of the increases was statistically significant (Figure 2A,B). However, KO(52del) zebrafish displays a significant increase in CO (33.1%, *p* = 0.03) and a trend of increase in SV (28%, *p* = 0.10) compared to HET siblings (Figure 2A,B), but unchanged HR and EF to their siblings (Figure 2C,D). We observed enlarged EDA in KO(52del) ventricles by 15.9% (*p* = 0.12) compared to WT and 21.5% (*p* = 0.02) to HET siblings (Figure 2E), suggestive of cardiac hypertrophy in KO(52del) adults. PWD signals displayed a heightened E wave (KO vs. WT *p* < 0.001; KO vs. HET *p* < 0.001; Figure 2F) and preserved A wave (Figure 2G) in KO(52del) hearts compared to their siblings, which ultimately led to an increase in E/A ratio (KO vs. WT *p* < 0.001; KO vs. HET *p* = 0.002; Figure 2H). However, the E‐wave deceleration times remained similar among the three genotypic groups (Figure 2I). The findings indicate that the increased CO could possibly be ascribed to increased SV resulted from enlarged ventricles in KO(52del). Notably, adult KO(52del) zebrafish displayed lower BW than their HET siblings (*p* = 0.03; Figure 2J). In line with KO(52del), inhibition of Hmox‐1 activity in WT zebrafish with ZnPPIX, a selective inhibitor of HMOX1,^36^ induced an enlargement of EDA by 19.4% (*p* = 0.04; Figure 2K) at 13 dpi. In addition, treatment with ZnPPIX increased CO by 35.4% (Figure 2L), although the change did not reach statistical significance. Taken together, deficiencies in the expression and activity of Hmox‐1 lead to cardiac hypertrophy and increased CO in adult zebrafish.

**FIGURE 3:**
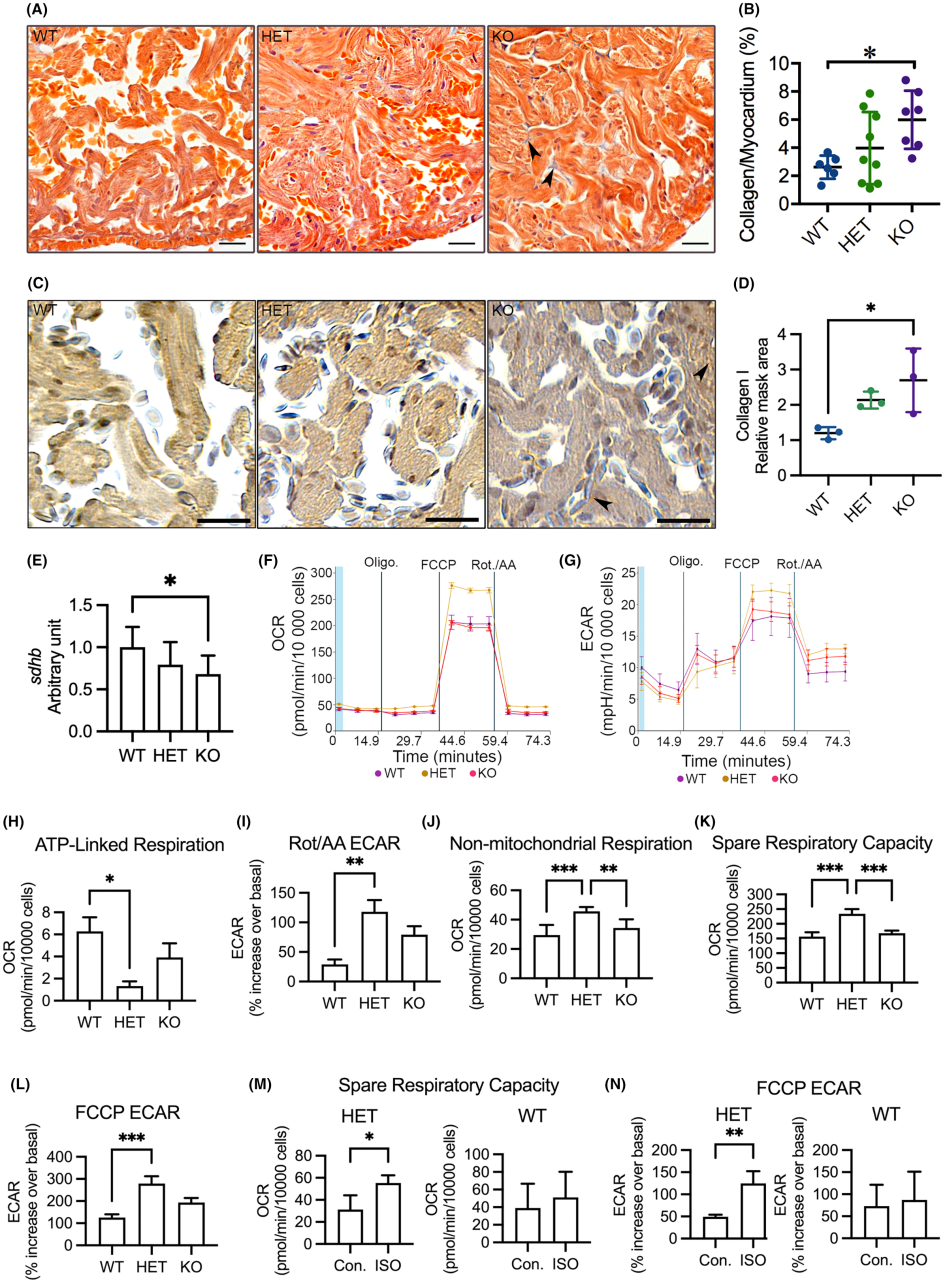
Analyses of myocardial interstitial fibrosis, cardiac OXPHOS gene expression and mitochondrial respiration in adult cardiomyocytes. (A) Representative images of acid fuchsin orange G (AFOG) staining of ventricular sections showing accumulation of collagen (blue) in the myocardium (orange). Arrowhead indicates accumulated collagen. (B) Quantification of AFOG staining indicating increased collagen accumulation in the myocardium in KO(52del). WT *n* = 6; HET(52del) *n* = 9; KO(52del) *n* = 7. (C) Representative images of immunohistochemical staining of ventricular sections for Collagen type I (brown). Arrowhead indicates Collagen type I‐positive signal. (D) Quantification of Collagen type I positive area relative to myocardium. WT *n* = 3; HET(52del) *n* = 3; KO(52del) *n* = 3. (E) RT‐qPCR analyses showing downregulation of OXPHOS complex II subunit *sdhb* in KO(52del) hearts compared to WT controls. *sdhb*, succinate dehydrogenase iron–sulfur subunit B; The graphs represent the quantification of two individual analyses of RNA extracts from pooled samples of two‐three hearts. Each analysis includes three replicates. WT *n* = 19; HET(52del) *n* = 19; KO(52del) *n* = 19. (F) Representative oxygen consumption (OCR) profile in zebrafish primary cardiomyocytes at basal respiration and after addition of oligomycin (Oligo.), carbonyl cyanide‐4 (trifluoromethoxy) phenylhydrazone (FCCP), followed by a combination of rotenone and antimycin A (Rot./AA). (G) Representative extracellular acidification (ECAR) profile in zebrafish primary cardiomyocytes at basal respiration and after addition of Oligo., FCCP and Rot./AA. (H) Cardiomyocytes from HET(52del) display a decline in the rate of respiration to drive mitochondrial ATP synthesis. (I) Cardiomyocytes from HET(52del) exhibit an increase in ECAR relative to the basal rate upon the addition of Rot./AA. (J) HET(52del) cardiomyocytes show increased non‐mitochondrial respiration. (K) HET(52del) cardiomyocytes show increased spare respiration capacity. (L) HET(52del) cardiomyocytes display increased ECAR relative to the basal rate upon the addition of FCCP. (M) Spare respiration capacity of HET(52del) and WT cardiomyocytes in response to ISO. (N) ECAR relative to the basal rate upon the addition of FCCP in HET(52del) and WT cardiomyocytes in response to ISO. (F–L) WT *n* = 15; HET(52del) *n* = 8; KO(52del) *n* = 7. M, N HET(52del) *n* = 15; WT *n* = 15. Data are presented as mean ± SD. One‐way ANOVA with Tukey adjustment for multiple comparisons in B, D, E and H–L. Two‐sample *t*‐test in M, N. *p < 0.05, **p < 0.01, ***p < 0.001. Scale bars: 20 μm.

Given that defects in the expression and activity of HMOX1 result in fibrosis and mitochondrial dysfunction in multiple metabolic tissues,^37^ we therefore explored whether deletion of *hmox1a* induces cardiac fibrosis and mitochondrial impairment in adults. Indeed, histological staining revealed an abundant accumulation of collagen in the myocardium in KO(52del) zebrafish (*p* = 0.02) compared to WT siblings (Figure 3A,B). Furthermore, immunohistochemistry staining indicated an increase in the expression level of Collagen type I in the myocardium in KO(52del) zebrafish (*p* = 0.04; Figure 3C,D).

Quantitative RT‐PCR indicated that the transcript of nuclear DNA‐encoded succinate dehydrogenase iron–sulfur subunit B (*sdhb*) of oxidative phosphorylation (OXPHOS) complex II markedly declined in *hmox1a*‐deficient hearts (KO vs. WT, *p* = 0.04; Figure 3E), whereas the mitochondrial DNA‐encoded cytochrome c oxidase subunit 1 (*mt‐co1*) of complex IV and NADH dehydrogenase subunit 1 (*mt‐nd1*) of complex I showed no significant alteration in KO(52del) compared to their siblings (Figure S2A,B). Concomitantly, mitochondrial DNA content was unchanged, suggesting that deletion of *hmox1a* has no major effect on mitochondrial abundance (Figure S2C). To further verify whether deletion of *hmox1a* could affect mitochondrial function, we isolated primary CMs from adult ventricles of each genotypic zebrafish and investigated real‐time cellular energy metabolism with Seahorse XF. Primary CMs isolated from WT and KO(52del) ventricles showed a subtle decrease in OCR upon addition of oligomycin to block mitochondrial ATP production, whereas CMs from HET(52del) were insensitive to oligomycin (Figure 3F), indicating a general low mitochondrial respiration in zebrafish primary CMs. However, OCR markedly increased when an artificial ATP demand was induced by FCCP and dropped when TCA cycle flux was shut down by rotenone and antimycin A (Figure 3F). In contrast, the primary CMs exhibited an increase in ECAR when oligomycin was added (Figure 3G), reflecting enhanced glycolytic turnover to meet energetic needs. ECAR was continually enhanced upon the addition of FCCP and decreased by rotenone and antimycin A (Figure 3G). Interestingly, CMs from HET(52del) exhibited a decrease in OCR (*p* = 0.02) devoted to ATP production (Figure 3H) and an increase in ECAR (*p* = 0.003) relative to basal rate upon addition of the respiratory inhibitors compared to WT controls (Figure 3I), indicating that haplodeficiency of *hmox1a* triggers non‐mitochondrial respiration for energy demand, possibly glycolysis, in cardiomyocytes. Consistently, non‐mitochondrial oxygen consumption was markedly increased in HET(52del) CMs compared with their siblings (HET vs. WT, *p* < 0.001; HET vs. KO, *p* = 0.006; Figure 3J). Furthermore, HET(52del) CMs displayed an increase in spare respiratory capacity (HET vs. WT, *p* < 0.001; HET vs. KO, *p* < 0.001; Figure 3K) and in ECAR (*p* = 0.001) relative to basal rate upon addition of FCCP (Figure 3L), suggesting a robust ability to respond to increased energy demand. Nevertheless, CMs from KO(52del) displayed no substantial alteration in mitochondrial respiration in comparison with WT controls. We further investigated the energy metabolism of HET(52del) CMs upon ISO treatment in comparison with WT CMs. ISO stimulated spare respiratory capacity (*p* = 0.02) and increased ECAR (*p* = 0.006) relative to basal rate upon addition of FCCP in HET(52del) CMs, but not in WT CMs (Figure 3M,N), suggesting a potential ability of HET(52del) CMs to meet energy demands possibly by increasing glycolysis in response to stress.

**FIGURE 4:**
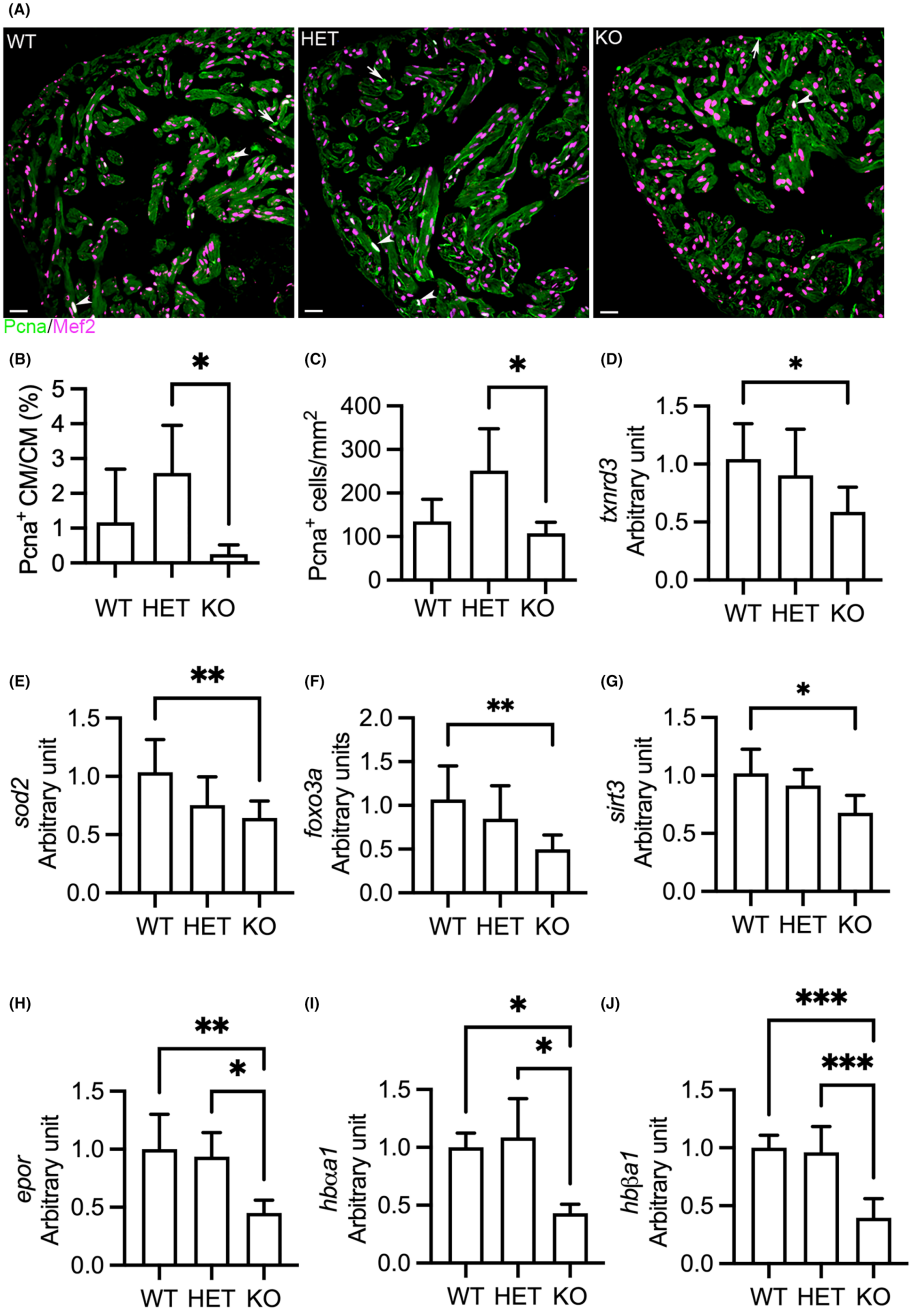
Deletion of *hmox1a* restrains cardiomyocyte proliferation and downregulates antioxidative genes and haemoglobin genes in adult zebrafish. (A) Representative immunohistochemical staining of Pcna (in green) and Mef2 (in meganne) in ventricular sections. Arrow indicates Pcna^+^ non‐cardiomyocytes. Arrowhead indicates Pcna^+^ cardiomyocytes. Pcna, proliferating cell nuclear antigen; Mef2, myocyte‐specific enhancer factor 2. (B) Quantification of Pcna^+^ cardiomyocytes relative to total cardiomyocytes. (C) Quantification of Pcna^+^ cardiac cells per mm^2^ ventricular area. Three hearts from each genotypic group were selected. Two sections from each heart were stained. The number of stain‐positive signals for Pcna and Mef2 from ventricular areas was quantified with ImageJ. (D–G) RT‐qPCR analyses indicating downregulation of antioxidative genes, *txnrd3* (D) and *sod2* (E), and the transcription regulators of antioxidative and antihypertrophic signalling, *foxo3a* (F) and *sirt3* (G), in KO(52del) hearts. *txnrd3*, *thioredoxin reductase 3*; *sod2*, *superoxide dismutase 2*; *foxo3a*, *forkhead box O3a*; *sirt3*, *sirtuin 3*. The graphs represent the quantification of two individual analyses of RNA extracts from pooled samples of two‐three hearts. Each analysis includes three replicates. WT *n* = 19; HET(52del) *n* = 19; KO(52del) *n* = 19. (H–J) RT‐qPCR analyses showing downregulation of erythropoietin receptor *epor* (H), and adult haemoglobin genes *hbαa1* (I) and *hbβa1* (J) in kidney lacking *hmox1a*. *epor*, *erythropoietin receptor*; *hbαa1*, *haemoglobin alpha adult‐1*; *hbβa1*, *haemoglobin beta adult‐1*. The graphs represent the quantification of two individual analyses of RNA extracts from pooled samples of two kidneys. Each analysis includes three replicates. WT *n* = 10; HET(52del) *n* = 10; KO(52del) *n* = 10. Data are presented as mean ± SD. One‐way ANOVA with Tukey adjustment for multiple comparisons. *p < 0.05, **p < 0.01, ***p < 0.001. Scale bars: 20 μm.

We next investigated cardiomyocyte proliferation. IHC indicated fewer Pcna^+^ cardiomyocytes in KO(52del) ventricles (*p* = 0.01) compared to their HET siblings, suggesting that deletion of *hmox1a* restrains cardiomyocyte proliferation (Figure 4A, B). Interestingly, HET(52del) exhibited increased cardiac cell proliferation compared to their KO(52del) siblings (*p* = 0.04; Figure 4C). Collectively, the data indicate that deletion of *hmox1a* increases cardiac output, induces cardiac hypertrophy and fibrosis, and restrains cardiomyocyte proliferation, suggestive of cardiac remodelling in adult KO(52del), whereas haplodeficiency of *hmox1a* triggers non‐mitochondrial respiration and stimulates cardiac cell proliferation.

The contradictory effect of HMOX1 on oxidative stress^22^, ^25^ prompts us to investigate the expression of cardiac genes involved in the antioxidative defence system. We found the downregulation of antioxidative genes,^38^ *thioredoxin reductase 3* (*txnrd3*) (*p* = 0.02; Figure 4D) and *superoxide dismutase 2* (*sod2*) (*p* = 0.007; Figure 4E), in KO(52del) hearts. The expression level of *forkhead box O3a* (*foxo3a*) (*p* = 0.005; Figure 4F), a transcription regulator of antioxidative signalling^39^ and a suppressor of myocardial hypertrophy,^38^ was also decreased in KO(52del) hearts. We further observed the downregulation of *sirtuin 3* (*sirt3*) (*p* = 0.02; Figure 4G), an activator of FOXO3,^40^ in KO(52del) hearts. The data indicate that deletion of *hmox1a* diminishes antioxidative defences and downregulates the redox‐sensitive antihypertrophic regulators at adult age, which probably leading to increased oxidative stress and activated redox‐sensitive hypertrophic signalling.

As HMOX1‐deficient patients develop haemolytic anaemia,^41^ we investigated erythropoiesis and haemoglobin synthesis in adults at the age of 5 months. Given that adult zebrafish erythropoiesis is maintained in the kidney marrow and regulated by erythroid transcription factors and erythropoietin signalling,^42^ we examined the expression of genes essential for erythropoiesis in the kidney by qPCR. We observed significant downregulation of *erythropoietin receptor* (*epor*), which binds erythropoietin to stimulate erythropoiesis, in KO(52del) compared to their siblings (KO vs. WT *p* = 0.007; KO vs. HET *p* = 0.01; Figure 4H). In addition, the expression of *haemoglobin alpha adult‐1* (*hbαa1*) (KO vs. WT, *p* = 0.05; KO vs. HET, *p* = 0.02) and *beta adult‐1* (*hbβa1*) (KO vs. WT, *p* < 0.001; KO vs. HET, *p* < 0.001) was also reduced in KO(52del) zebrafish (Figure 4I, J). On the other hand, the expression of *erythropoietin a* (*epoa*) was unchanged (Figure S2D). Furthermore, the expression of transcription regulators in erythropoiesis, *krüppel‐like factor d* (*klfd*) and *GATA binding protein 1a* (*gata1a*), was not significantly different in KO(52del) compared to their siblings (Figure S2E,F). These results suggest that the deletion of *hmox1a* impairs haemoglobin synthesis in adults, which may contribute to cardiac remodelling.

### 3.4 HET(52del) larvae display increased cardiac output in response to hypoxia

**FIGURE 5:**
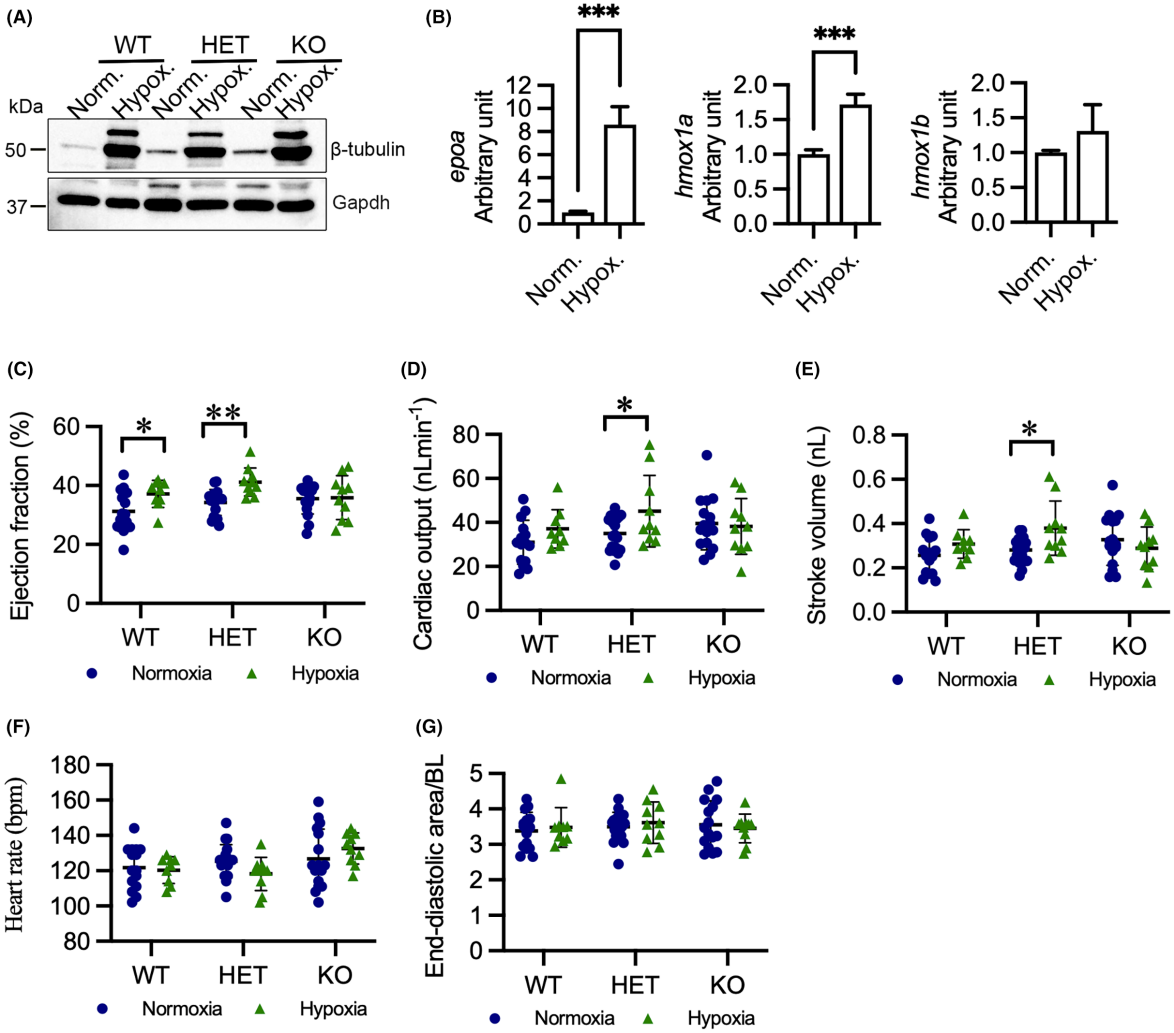
Hypoxia enhances cardiac output in HET(52del) larvae. (A) Representative immunoblots of β‐tubulin and Gapdh in WT, HET(52del), and KO(52del) at normoxic and hypoxic (3% O_2_ for 24 h) conditions. Gapdh serves as an internal control. Norm. normoxia; Hypox. Hypoxia. (B) RT‐qPCR analyses indicate that hypoxia induces upregulation of *epoa* and *hmox1a*, but not *hmox1b*, in WT larvae. (C–G) Hypoxia enhances ejection fraction (C) in WT and HET(52del) larvae, and cardiac output (D) and stroke volume (E) in HET(52del) larvae, but not in KO(52del), compared to normoxia. Hypoxia has no effect on heart rate (F) or diastolic area (G) in the three genotypic groups. WT, normoxia *n* = 15, hypoxia *n* = 9; HET(52del), normoxia *n* = 19, hypoxia *n* = 10; KO(52del), normoxia *n* = 16, hypoxia *n* = 10. Data are presented as mean ± SD. Two‐sample *t*‐test. *p < 0.05, **p < 0.01, ***p < 0.001.

HMOX1 exerts cardioprotective effects after injury.^11^ Thus, we wondered whether disruption of *hmox1a* deteriorates cardiac dysfunction in response to injury in zebrafish. Hypoxia, which can occur in both physiological and pathological conditions, has significant implications for cardiovascular disease.^43^ As Hmox1 plays a role in controlling cardiac function in response to hypoxia,^44^ we exposed larvae to a low oxygen (hypoxia) environment (3% O_2_). Given that hypoxia induces ß‐tubulin gene expression mediated by hypoxia‐inducible factor (HIF),^45^ we analysed the expression of ß‐tubulin in larvae exposed to normal oxygen (normoxia) or to 3% O_2_ for 24 h. The protein level of ß‐tubulin substantially increased in larvae exposed to 3% O_2_ (Figure 5A). We also observed the transcriptional upregulation of *epoa* (*p* < 0.001) and *hmox1a* (*p* < 0.001), which are target genes of Hif‐1α, in WT larvae exposed to 3% O_2_ (Figure 5B). The data indicate induction of hypoxia in vivo in larvae exposed to 3% O_2_. Notably, hypoxia had no substantial effect on the expression of *hmox1b* (Figure 5B).

We next evaluated cardiac function in normoxic and hypoxic larvae. Hypoxia caused elevated EF in WT and HET(52del) larvae (WT *p* = 0.05; HET *p* = 0.001), but not in KO(52del) larvae (Figure 5C). In addition, HET(52del) larvae exhibited enhanced CO (*p* = 0.02) and SV (*p* = 0.02) when exposed to hypoxia, whereas their WT and KO siblings showed no significant change (Figure 5D,E). Moreover, hypoxia had no impact on HR in either WT or KO but tended to reduce HR (*p* = 0.06) in HET (Figure 5F). Neither the mutants nor WT larvae exhibited changed EDA in response to hypoxia (Figure 5G). Collectively, the data suggest that deletion of *hmox1a* fails to induce cardiac compensation to overcome the lack of oxygen, whereas haplodeficiency of *hmox1a* boosts cardiac contractility and output in response to hypoxia.

### 3.5 HET(52del) adults deteriorate cardiac function in response to ISO treatment

**FIGURE 6:**
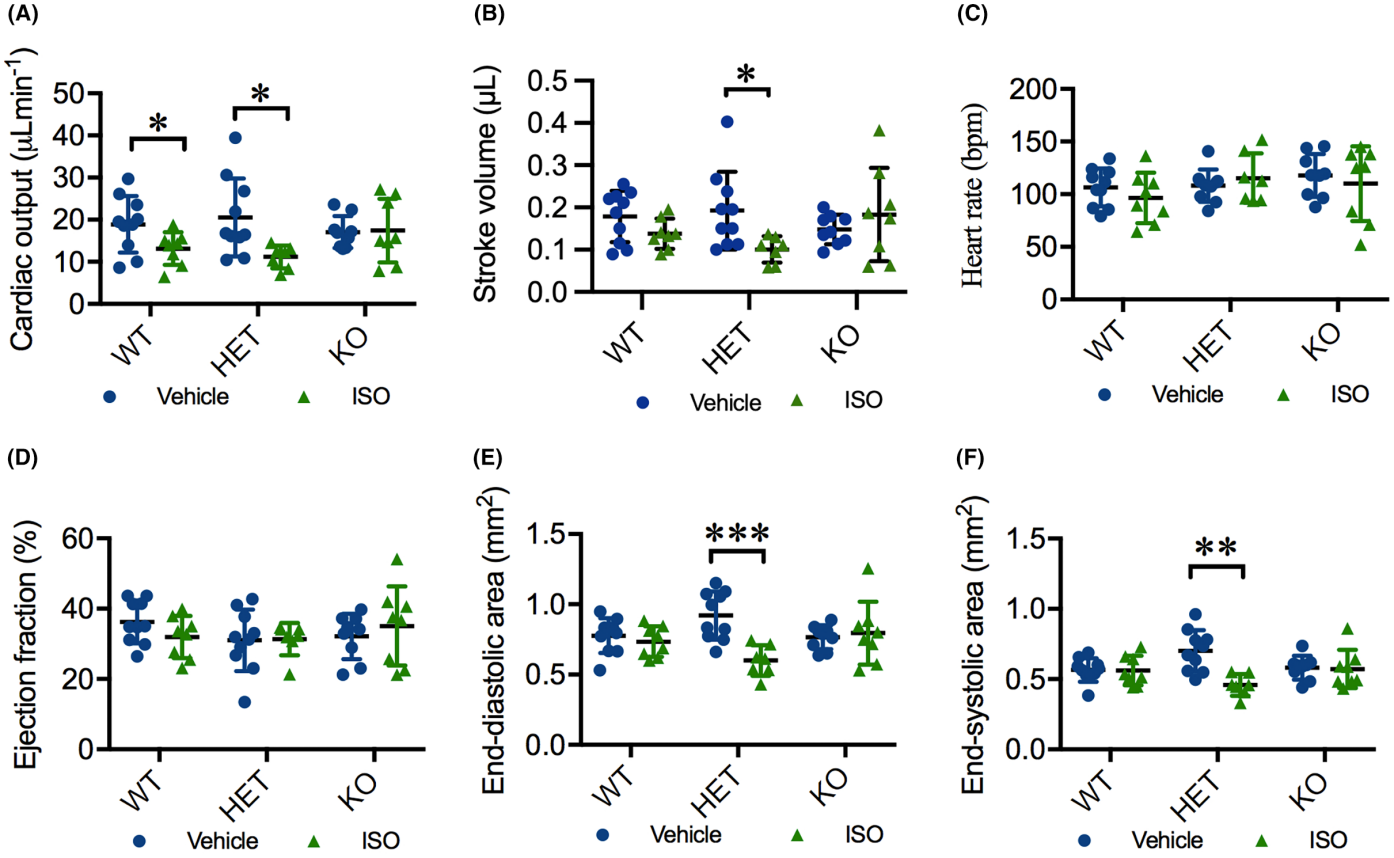
ISO deteriorates cardiac function in HET(52del) adults. (A) ISO treatment results in reduced cardiac output in WT and HET(52del), but not in KO(52del), compared to vehicle‐treated controls. (B) ISO treatment leads to reduced stroke volume in HET(52del), not in WT and KO(52del) compared to vehicle‐treated controls. (C, D) ISO treatment has no significant effect on heart rate (C) or ejection fraction (D) in the three genotypic groups compared to respective vehicle controls. (E, F) ISO treatment leads to reduced end‐diastolic (E) and end‐systolic area (F) in HET(52del), not in WT and KO(52del) compared to vehicle‐treated controls. WT, vehicle *n* = 10, ISO *n* = 8; HET(52del), vehicle *n* = 10, ISO *n* = 7; KO(52del), vehicle *n* = 9, ISO *n* = 8. Data are presented as mean ± SD. Two‐sample *t*‐test. * *p* < 0.05, ** *p* < 0.01, *** *p* < 0.001.

A high dosage of ISO enhances myocardial function and eventually induces myocardial dysfunction in rats, representing an established ischaemic heart failure model.^33^ Accordingly, we investigated whether deletion of *hmox1a* exacerbates cardiac dysfunction in response to ISO in zebrafish. ISO markedly boosts cardiac function in early larvae in all three genotypic groups (Figure S3). We treated WT adult zebrafish with ISO at a dosage of 150 mg/kg or 75 mg/kg and observed cardiac response to 150 mg/kg, but not 75 mg/kg, ISO (Figure S4). Thus, a high dosage of ISO (150 mg/kg) was administered to the adult zebrafish. We found reduced CO in WT (*p* = 0.05) and HET(52del) (*p* = 0.02) zebrafish in comparison with vehicle‐treated controls but had no effect in the KO(52del) siblings (Figure 6A). In addition, HET(52del) zebrafish also displayed reduced SV (*p* = 0.02) in response to ISO treatment, whereas WT and KO(52del) siblings showed no obvious alteration in SV (Figure 6B). ISO treatment had no substantial effect on HR and EF in either WT or the mutants (Figure 6C, D). However, ISO‐treated HET(52del) displayed smaller EDA (*p* < 0.001) and ESA (*p* = 0.001) compared to vehicle‐treated controls, while WT and KO siblings showed no obvious alteration in the cardiac area (Figure 6E, F). Taken together, the deletion of *hmox1a* fails to induce a cardiac response to ISO in adult zebrafish, but the haplodeficiency of *hmox1a* deteriorates cardiac function.

**FIGURE 7:**
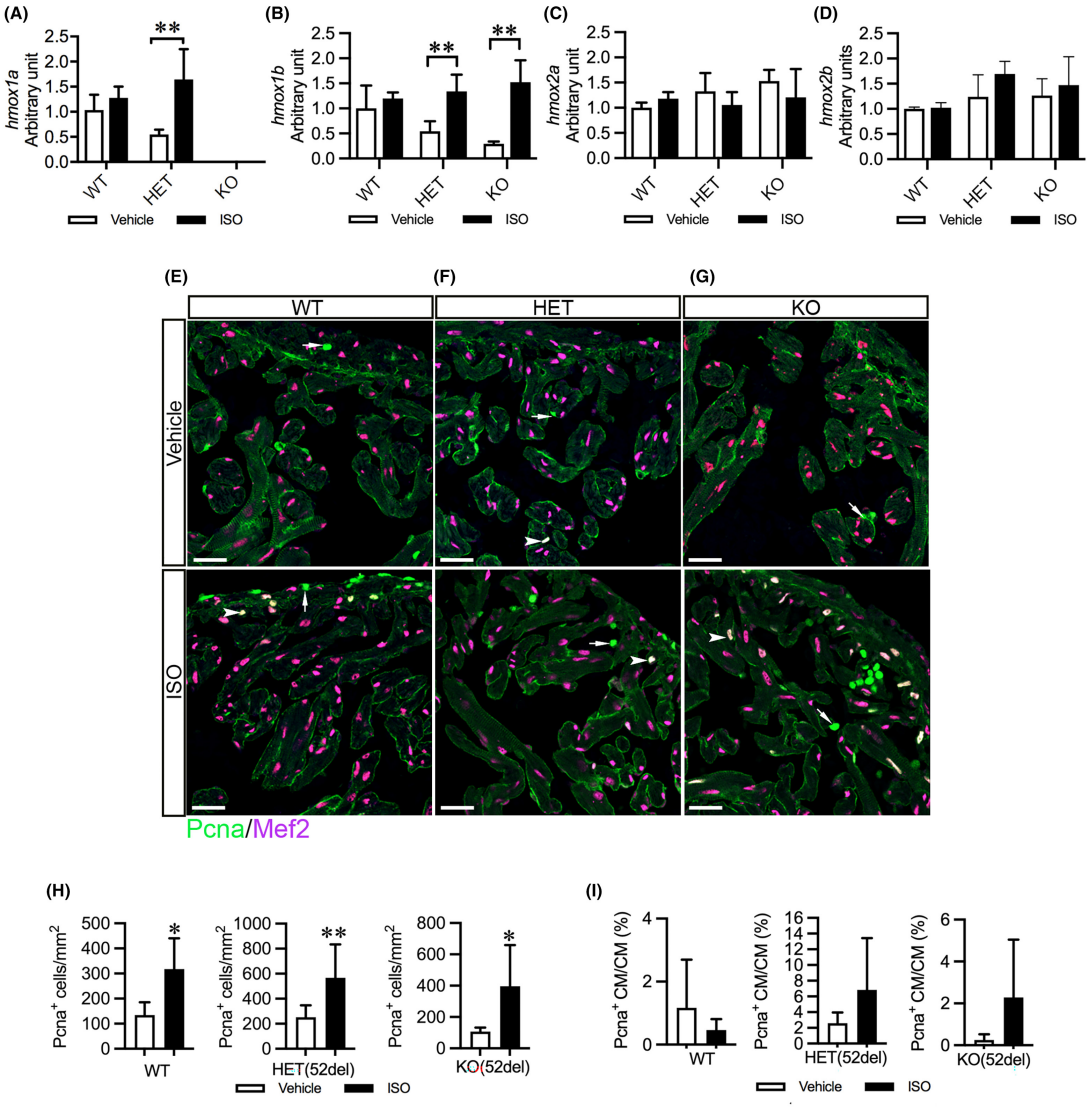
ISO upregulates cardiac *hmox*1 paralogs and increases cardiac cell proliferation in HET(52del) adults. (A–D) RT‐qPCR analyses of the expression of *hmox*1 homologues in ISO‐induced zebrafish hearts compared to vehicle‐treated controls. The results are presented as fold change compared to vehicle‐treated WT hearts. WT, vehicle *n* = 6, ISO *n* = 4; HET(52del), vehicle *n* = 6, ISO *n* = 4; KO(52del), vehicle *n* = 5, ISO *n* = 4. (E–G) Representative immunohistochemical staining of Pcna (in green) and Mef2 (in meganne) in ventricular sections from vehicle‐ or ISO‐treated zebrafish in each genotypic group. Arrow indicates Pcna^+^ non‐cardiomyocytes. Arrowhead indicates Pcna^+^ cardiomyocytes. (H, I) Quantification of Pcna^+^ cardiac cells per mm^2^ ventricular area (H) and Pcna^+^ cardiomyocytes relative to cardiomyocytes (I). Three hearts from each genotypic group were chosen. Two sections from each heart were stained. The number of stain‐positive signals for Pcna and Mef2 from ventricular areas was quantified with ImageJ. Data are presented as mean ± SD. Two‐sample *t*‐test. *p < 0.05, **p < 0.01.

To explore the potential mechanism underlying the distinct cardiac impact on heterozygous and homozygous *hmox1a* mutants in response to cardiac stress, we quantitatively analysed the expression of *hmox1a* in ISO‐treated larval and adult hearts compared with that in vehicle‐treated controls. As expected, the *hmox1a* transcript was absent in KO(52del) larvae treated either with ISO or vehicle (Figure S3F). In addition, ISO had no significant effect on the expression of *hmox1a* in either WT or HET(52del) larvae (Figure S3F). Intriguingly, ISO treatment resulted in profound upregulation of *hmox1a* in HET(52del) adult hearts (ISO vs. vehicle, *p* = 0.002), but not in WT controls (Figure 7A). We further observed a substantial upregulation of *hmox1b* in KO(52del) (ISO vs. vehicle, *p* = 0.002) and HET(52del) (ISO vs. vehicle, *p* = 0.004) hearts in response to ISO treatment, but no alteration in WT hearts (Figure 7B). However, ISO had no effect on the expression of *hmox2a* and *hmox2b* in either WT or mutant hearts (Figure 7C,D). These data suggest that upregulation of *hmox1b* exerts a counter‐protective effect in *hmox1a* KO hearts, whereas excessive expression of both HMOX1 paralogs elicits a detrimental effect in *hmox1a* HET hearts in response to ISO. In addition, IHC indicated that ISO treatment induced a significant increase in cell proliferation in the ventricles of all three genotypic groups (WT *p* = 0.01, HET *p =* 0.002, KO *p =* 0.02; Figure 7E–H). In contrast, the number of Pcna^+^ cardiomyocytes showed no significant difference upon ISO treatment in the three genotypic groups (Figure 7E–G,I). However, Pcna^+^ cardiac cells were mostly distributed in the compact layer of the ventricular wall in ISO‐treated WT (Figure 7E), whereas positive signals often appeared in the trabecular layer in ISO‐treated mutants (Figure 7F,G).

## 4 DISCUSSION

We generated a mutation allele of *hmox1a*, *hmox1a* (52del), with a premature termination codon in exon 3 that possibly induces nonsense‐mediated mRNA decay using CRISPR. The deficiency of *hmox1a* did not induce the expression of its paralog *hmox1b*, or its isoforms, *hmox2a and hmox2b*. Neither homozygous nor heterozygous larvae displayed excess mortality or gross morphological abnormalities. Although they were adult viable, we noticed a short lifespan of homozygous mutants and a reduced spawning frequency in heterozygous adults. Unlike our findings, a previous study demonstrated that a minority of zebrafish *hmox1a* ^vcc42/vcc42^ mutants displayed abnormal morphology at 3 dpf that progressed to lethality within 14 dpf.^28^ However, *hmox1a* ^vcc42/vcc42^ adult zebrafish showed no gross morphological abnormalities compared to their WT siblings. Genetic editing in the *hmox1a* locus led to a significant induction of *hmox1b*, which was even higher in abnormal embryos but not of *hmox2a* or *hmox2b*, in both *hmox1a* ^vcc42/vcc42^ mutant and *hmox1a* crispants.^28^ The mutant was generated by targeting the start code in exon 2 via TALENs, resulting in a deletion of exon 2 and a predicted mutant protein lacking 36 N‐terminal amino acid residues. The different phenotype between *hmox1a* (52del) and *hmox1a* ^vcc42/vcc42^ mutant lines may be partially ascribed to the residual peptide translated from the *hmox1a* ^vcc42/vcc42^ transcript and the upregulation of *hmox1b*.

Consistent with HMOX1 deficiency in mammals, *hmox1a* (52del) zebrafish developed interstitial fibrosis in the myocardium, dysregulated haemoglobin synthesis and reduced body weight compared to HET siblings. We demonstrate that deletion of *hmox1a* increases cardiac output in larval and adult zebrafish and induces cardiac hypertrophy in adult zebrafish. The different impact of the deletion of *hmox1a* in larvae versus adults could be because larvae are developing and can compensate for the deficiency of *hmox1a*, whereas the chronic absence of *hmox1a* leads to decompensation and remodelling with age. Similarly, patients with HMOX1 deficiency were initially normal until the onset of symptoms and rapid deterioration.^41^ Similar to our findings, downregulation of Hmox1 with morpholinos results in a significant enlargement of the ventricle in 4 dpf zebrafish larvae.^44^ However, unlike *hmox1a* mutant larvae, Hmox1 morphants exhibited no difference in CO despite significantly higher HR compared to controls. The discrepancy between *hmox1a* mutant and morphant phenotypes could be ascribed to a more severe effect of *hmox1a* deletion in comparison with the transient decline of Hmox1. An increase in cardiac output and enlargement of the ventricle have also been observed in phenylhydrazine hydrochloride‐induced anaemic rats and zebrafish.^46^, ^47^ Phenylhydrazine hydrochloride induces oxidative haemolysis of red blood cells and reduction of haemoglobin, ultimately resulting in anaemia. Anaemia consequently triggers cardiac remodelling by increasing cardiac output and ventricular stretch.^46^ Indeed, we observed the downregulation of genes encoding haemoglobin in KO(52del). We also demonstrate that deletion of *hmox1a* downregulates the genes encoding antioxidative enzymes and transcription regulators that suppress cardiac hypertrophy in KO(52del). A previous study indicated that inhibition of HMOX1 activity increases oxidative stress, aggravating cardiac hypertrophy.^25^ Accordingly, the elevated cardiac output and cardiac hypertrophy observed in *hmox1a*‐deficient zebrafish may be ascribed to anaemia, decreased antioxidative defences and impaired redox‐sensitive anti‐hypertrophic signalling.

Given that HMOX‐1 exerts cardioprotective effects in response to injury,^11^ we expected that the deletion of *hmox1a* would fail to protect against cardiac injury. We observed elevated cardiac contractility in WT and HET(52del) larvae when exposed to hypoxia. This could be ascribed to the less availability of oxygen in the environment, which triggers the cardiac response to overcome the demand for oxygen. This is in line with a previous study showing that genetically hypoxic larvae (*vhl* ^*−/−*^)^48^ displayed higher cardiac output, especially at 6 dpf, than WT controls.^49^ HET(52del) larvae exhibited significantly higher CO when exposed to hypoxia. This could be due to the robust ability of HET(52del) CMs to respond to the increased energy demand and stress. Similar to our finding, the downregulation of Hmox1 by morpholino also induced higher CO level in zebrafish larvae when exposed to hypoxia.^44^ Strikingly, KO(52del) larvae failed to increase cardiac contractility and output when exposed to hypoxia. This suggests that hypoxia boosts cardiac function dependent on Hmox1a. Indeed, hypoxia induced the transcriptional upregulation of *hmox1a* in WT larvae.

Exposure to a high dosage of ISO caused a significant decrease in CO in WT and HET but not in KO(52del) adult zebrafish in the present study, suggesting that a lack of *hmox1a* prevented ISO from exerting its effect on specific sympathetic nerve stimulation of the heart. In support of our notion, previous studies demonstrated that ISO failed to elicit its beneficial effects on the treatment of sepsis when HMOX1 expression was suppressed by ZnPPIX, an HMOX1 inhibitor.^50^, ^51^ In addition, Hmox1 morphant larval hearts were insensitive to adrenaline under hypoxic conditions, whereas adrenaline increased HR in sham‐treated larvae.^44^ We further found that ISO substantially stimulates the expression of *hmox1a* and *hmox1b* in HET(52del) zebrafish adult hearts. This is in good agreement with previous reports that ISO induced HMOX1 expression in mouse leukaemic monocyte‐macrophages and in heart and lung tissues in septic mice in a concentration‐dependent manner.^50^ Induction of HMOX1 has been associated with chronic viral myocarditis and cardiac hypertrophy in mice.^22^ Septic, critically ill patients carrying a short GTn allele in the *HMOX1* promoter region have high plasma HMOX1 concentration and are associated with the development of acute kidney injury.^52^ Excessive expression of *hmox1a* and *hmox1b* in HET(52del) hearts compared to WT controls may exert a detrimental effect on cardiac function in response to ISO.

Decrease of mitochondrial respiration or promotion of glycolytic metabolism is critical for cardiomyocyte proliferation and heart regeneration in zebrafish.^53^, ^54^ In line with this, heterozygous *hmox1a*(52del) exhibited increased non‐mitochondrial oxygen consumption in adult cardiomyocytes and heightened cardiac cell proliferation compared to their WT and KO siblings, indicating cardiac reprogramming from a high‐metabolic, non‐proliferative state to a low‐metabolic, proliferative state. Furthermore, they display a robust ability to the increased energy demand and in response to stress. Whether such cardiac reprogramming elicits a protective or adverse effect on cardiac function and structure needs to be studied further.

The presence of duplicate copies of mammalian *HMOX1* and functional compensation in zebrafish can be a limitation of our study. *Hmox1a* appears to be necessary for normal development in zebrafish, and its expression is responsive to oxidative stress, suggesting a conserved physiological function to mammals.^28^ Although knockout of *hmox1a* cannot completely delete Hmox1 protein, lowering the level of Hmox1 predisposes to cardiac remodelling with age. Thus, *hmox1a* knockout zebrafish could serve as a model resembling human long GTn *HMOX1* polymorphism, which leads to lower HMOX1 protein levels and predisposes to cardiovascular disease.

In conclusion, *hmox1a* deficiency boosts cardiac output and induces cardiac hypertrophy in adult zebrafish. The changes in cardiac function and structure could be partially ascribed to restrained cardiomyocyte proliferation, myocardial interstitial fibrosis, impaired haemoglobin synthesis, decreased antioxidative defences and inactive anti‐hypertrophic signalling. Intriguingly, the haplodeficiency of *hmox1a* in adult zebrafish triggers upregulation of both *hmox1a* and *hmox1b* and aggravates cardiac dysfunction in response to ISO stimulation. Our data indicate that HMOX1 homeostasis is essential for maintaining cardiac function and prompting cardioprotective effects, suggesting that the induction of HMOX1 for cardiac therapeutics needs to be leveraged.

## AUTHOR CONTRIBUTIONS

**Hong Wang:** Formal analysis (equal); investigation (equal); project administration (equal); visualization (lead); writing – original draft (lead); writing – review and editing (equal). **Juuso Siren:** Conceptualization (equal); formal analysis (equal); investigation (equal); project administration (equal). **Sanni Perttunen:** Formal analysis (equal); investigation (equal); project administration (equal). **Katariina Immonen:** Formal analysis (equal); investigation (equal). **Yu‐chia Chen:** Methodology (equal). **Suneeta Narumanchi:** Investigation (equal). **Riikka Kosonen:** Investigation (equal). **Jere Paavola:** Conceptualization (equal); writing – review and editing (equal). **Mika Laine:** Conceptualization (equal). **Ilkka Tikkanen:** Conceptualization (equal); funding acquisition (equal); supervision (equal). **Päivi Lakkisto:** Conceptualization (equal); funding acquisition (equal); supervision (equal); writing – review and editing (equal).

## FUNDING INFORMATION

The work was supported by grants from the Finnish Cultural Foundation (HW, SN, PL), the Finnish Foundation for Cardiovascular Research (HW, IT, PL), the Aarne Koskelo Foundation (HW, SN, IT, PL), Ida Montin Foundation (SN), the Finnish Foundation for Laboratory Medicine (PL), Finska Läkaresällskapet (IT, PL), the Liv och Hälsa Foundation (IT, PL), the Finnish Society of Clinical Chemistry (PL), Päivikki and Sakari Sohlberg Foundation (PL), and Finnish state funding for university‐level research (IT, PL). Open access funding was provided by University of Helsinki.

## CONFLICT OF INTEREST STATEMENT

The authors declare that there is no conflict of interest associated with this manuscript.

## Supporting information


Data S1.

## Data Availability

The datasets used and/or analysed during the current study are available from the corresponding author upon reasonable request.
