# Supplementary material for: Deficiency of heme oxygenase 1a causes detrimental effects on cardiac function
Source: J Cell Mol Med. 2024 Mar 20;28(7):e18243. doi: 10.1111/jcmm.18243 (PMC10955162; doi:10.1111/jcmm.18243)
Supplement: Supplementary file 1 — Data S1. [file JCMM-28-e18243-s001.docx]

**Deficiency of *Heme Oxygenase 1a* Causes Detrimental Effects on Cardiac Function**

**Wang *et al.***

**Supplemental Materials and Methods**

**Generation of zebrafish *hmox1a*mutants**

Zebrafish of the Turku line has been maintained in a recirculating aquatic system at 28°C with a 14:10 hours light: dark cycle in the zebrafish core facility at the University of Helsinki for more than fifteen years. Animal experiments were approved by the Regional Government Office of Southern Finland in agreement with the ethical guidelines of the European Union (ESAVI/4131/04.10.07/2017; ESAVI/16286/2020). The CRISPR/Cas9 targeted mutagenesis was performed as previously described [1] . Briefly, we designed a CRISPR/Cas9-genome edited mutation by targeting exon 3 of zebrafish *hmox1a* (NM_001127516.1). The target site, 5’-GGAGGCTCTGGGGCAGGACTTGG-3’, was selected by using ZiFiT Targeter software (http://zifit.partners.org/) without predicted off-targeting site. The single guide RNA (sgRNA) was synthesized using the MAXIscript T7 kit (Life Technologies, Carlsbad, CA, USA) according to Varshney *et al*. [2] . The sequence of *hmox1a* gRNA crRNA:tracrRNA was 5’-gcgTAATACGACTCACTATAGGAGGCTCTGGGGCAGGACTGTTTTAGAGCTAGAAATAGC-3’. The Cas9 mRNA was synthesized with the plasmid pMLM3613 encoding Cas9 nuclease (Addgene, plasmid #42251) and the mMESSAGE T7 ULTRA kit (Life Technologies). Approximately 200 embryos were co-injected with 600 pg Cas9 mRNA and 26 pg gRNA at the one-cell stage. The target locus was amplified by PCR from the genomic DNA obtained from a 3 days post fertilization (dpf) tail clip of normal developing embryos. The mutated alleles were identified via high-resolution melting (HRM) analysis (Roche Diagnostics GmbH, Mannheim, Germany) and Sanger sequencing by comparison with the wild-type sequence. Only normally developed embryos with somatic mutations were raised to adulthood as founders. Ten founders (F0) carrying a 52 bp deletion in exon 3 of *hmox1a* were outcrossed to the Turku wild type to obtain F1 zebrafish heterozygous for *hmox1a* mutations. The F1 *hmox1a* heterozygous adults were subsequently inbred to obtain F2 wild type (WT) (*hmox1a*^+/+^), heterozygous (HET) (*hmox1a*^+/-^) and homozygous (KO) (*hmox1a*^-/-^) zebrafish. HET mutants of F2 or later generations were inbred to generate WT, HET, and KO embryos used in this study.

**Genotyping**

We genotyped either adult zebrafish or 3 dpf embryos at each generation as described previously [2, 3] . Briefly, genomic DNA was extracted from individual tail clippings with the lysis buffer (10 mM Tris-HCl, pH 8.3, 50 mM KCl, 0.3% Tween-20, and 0.3% NP-40) at 98°C for 10 minutes, followed by incubation on ice for 10 minutes. Proteinase K (20 mg/ml) was added to remove protein by incubating the extract at 55°C overnight and inactivated at 98°C for 10 minutes. Then, 1 μL of the genomic DNA extract was used as the template to amplify the target regions with LightCycler 480 HRM master mix (Roche Diagnostics) and the corresponding primers (Table S1) on a LightCycler 480 instrument (Roche Diagnostics). HRM curve was acquired and analysed using the LightCycler 480 gene scanning program (version 1.5, Roche Diagnostics) according to the manufacturer’s instructions. Each genotype displayed a distinct melting curve and embryos with analogous melting curves were characterized as the same genotype.

**Cardiac function of zebrafish larvae**

The cardiac function of larvae of mixed gender at 5-6 dpf was analysed as described earlier [4] . Briefly, larvae were equilibrated at room temperature (RT) and mounted in 3% methylcellulose. Live videos of beating hearts were recorded with an Olympus IX70 microscope (Olympus, Tokyo, Japan) equipped with a Hamamatsu ORCA-Flash 4.0 CMOS camera and HCImage software (Hamamatsu Photonics, Shizuoka, Japan). Videos were analysed with ImageJ (Bethesda, MD, USA). Ventricular width (*a*) and length (*b*) in the end diastole and systole were measured to obtain cardiac dimensions. Ventricular area (VA) was calculated using the formula, $VA=\pi\left( {ab}/2 \right),$and normalized to the body length. Ventricular volume was calculated by the following formula: $ventricular volume=a^{2}\times b\times0.523$. Ejection fraction (EF) was calculated as the difference between end-diastolic volume (EDV) and end-systolic volume (ESV) divided by EDV: $EF=\left[ \left( EDV-ESV \right)/{EDV} \right]\times100$. Stroke volume (SV) was calculated by subtracting ESV from EDV: $SV=EDV-ESV$. Cardiac output (CO) was calculated by multiplying SV with heart rate (HR): $CO=SV\times HR$. For each heart, five diastoles and systoles were analyzed.

**Echocardiography of adult zebrafish**

Echocardiography of adult zebrafish of mixed gender was performed with Vevo 2100^®^ Image System and Vevo Imaging Station (VisualSonics, Amsterdam, Netherlands) equipped with a high-frequency transducer (MS700, 30-70MHz) as described previously [5] . In brief, fish were equilibrated at RT and anesthetized with 0.02% tricaine in system water kept at RT overnight. Echocardiographic images were acquired in a longitudinal axis (LAX) view to record B-mode videos and in a short axis (SAX) view to obtain pulsed-wave Doppler (PWD) signals. Image acquisition was typically completed within 3-4 minutes of anaesthesia induction and no mortality was observed. After echocardiography, fish were placed into a tank filled with fresh system water, and usually recovered within 20 seconds.

Image analysis was conducted offline with the Vevo Lab™ analysis software (VisualSonics) by experienced personnel as described recently [6] . Briefly, ventricular longitudinal diameters from five consecutive cardiac cycles were obtained from B-mode images by measuring the perpendicular distance from the base to the apex at end-diastole (EDD) and end-systole (ESD), respectively. The ventricular area was defined as the area within the inner border of the compact myocardium at end-diastole (EDA) and end-systole (ESA). Ventricular EDV and ESV were then calculated based on the corresponding diameters and areas using the formula, $EDV=\text{8}\times{{EDA}^{2}}/{3\pi\times EDD}$ or $ESV=8\times{{ESA}^{2}}/{3\pi\times ESD}$. EF, SV, and CO were determined from EDV and ESV. For assessment of diastolic function, the maximal velocity of blood inflow across the atrioventricular valve during early diastole (E wave) and atrial systole (A wave) as well as the deceleration time of E wave were derived from PWD signals. HR was also obtained from the PWD image.

**Hypoxia exposure in zebrafish larvae**

Zebrafish larvae at 4 dpf were individually placed in 500 μL E3 medium in 48-well plate and exposed to hypoxia (3% of O_2_, OxyCycler, BioSpherix, NY, USA) at 28°C for 24 hours in a Heracell VIOS 160i incubator (Thermo Fisher Scientific Inc., Waltham, MA, USA). To create and maintain oxygen conditions, nitrogen gas was bubbled into the sealed incubator. Oxygen concentrations were measured with a Fibox 3 fiber optic oxygen probe and transmitter (PreSens Precision Sensing GmbH, Regensburg, Germany). Half of the embryos were placed under hypoxic condition and half were placed under normoxic condition. This resulted in six treatment groups: WT normoxia, WT hypoxia, HET normoxia, HET hypoxia, KO normoxia, and KO hypoxia. After 24-hour exposure, larvae were immediately subjected to either cardiac function analysis or snap-frozen.

**Zn(II) Protoporphyrin IX treatment in adult zebrafish**

Wild-type adult zebrafish were anesthetized with 0.02% tricaine in system water and i.p. injected with Zn(II) protoporphyrin IX (ZnPPIX, Frontier Scientific, Logan, UT, USA) at a dose of 5 mg/kg body weight on days 1 and 7. Control zebrafish received saline. The handling of ZnPPIX was performed in the dark due to its sensitivity to light. Echocardiographic examinations were conducted before injection and at 13 dpi.

**Isoproterenol treatment in larval and adult zebrafish**

Zebrafish embryos received 300 µM of isoproterenol (ISO, Sigma-Aldrich, Munich, Germany) at 2 dpf through E3 medium (5.0 mM NaCl, 0.4 mM CaCl2, 0.3 mM MgSO4 and 0.2 mM KCl) for 4 days. Larval cardiac function was monitored at 6 dpf. Adult zebrafish of mixed gender were anaesthetized with 0.02% tricaine in system water and i.p. injected with a single high dose of ISO at 150 mg/kg body weight [7] . Fish from each genotype were randomly divided into two groups based on body size: control and ISO. Control zebrafish received saline. ISO was dissolved in DMSO and diluted in saline to an injection volume of 0.3 to 0.6 μL. At 7 dpi, an echocardiographic examination was conducted. At 8 dpi, fish were anaesthetized with 0.02% tricaine in system water and hearts were dissected, fixed in 4% paraformaldehyde followed by embedding in paraffin, or three individual hearts were pooled and snap-frozen in dry ice and stored at -80°C.

**Primary cardiomyocytes isolation**

Primary cardiomyocytes (CMs) were isolated and purified from adult zebrafish ventricles of KO(52del), HET(52del), and WT zebrafish as previously described [8] . Briefly, fish were anaesthetized with 0.02% tricaine. The ventricles were dissected, cleaned in perfusion buffer (1× PBS plus 10 mM HEPES, 30 mM taurine, 5.5 mM glucose and 10 mM 2,3-butanedione monoxime), and digested in freshly prepared digestion buffer (1x perfusion buffer, 12.5 µM CaCl_2_, 5 mg/ml collagenase II, 5 mg/ml collagenase IV) at 32°C for 2 hours in a thermomixer. The dissociated cells were collected by centrifugation at 250 g for 5 minutes at 4°C and gradually exposed to increasing calcium concentrations to the physiological level of 1 mM at 4°C to obtain high-purity cardiomyocytes. Purified cardiomyocytes were resuspended in plating medium (MEM, 2 mM GlutaMAX, 5% FBS, 5 mM 2,3-butanedione monoxime, 1% penicillin/streptomycin, 0.2% normocin) and seeded in poly L-lysine-coated cell culture plates (Agilent Seahorse XF96 Cell Culture Microplates, Santa Clara, CA, USA) at 10 000 cells/well. The cells were cultured at 28°C in 5% CO_2_ and the medium was changed to plating medium without 2,3-butanedione monoxime after 24 hours.

**Cardiomyocyte energy metabolism**

The oxygen consumption rate (OCR) and the extracellular acidification rate (ECAR) in 4-day primary CMs were measured with an XF Mito Stress Test Kit (Agilent) by using a Seahorse XF^e^96 analyser (Agilent) following the manufacturer’s instructions. Prior to assay, the culture medium was replaced with XF assay medium DMEM supplemented with 10 mM glucose, 2 mM glutamine, and 1 mM pyruvate. Cells were equilibrated for 1 hour at 28°C in a CO_2_-free incubator. During the assay, basal OCR and ECAR were measured, followed by sequential injections of 2 µM oligomycin, 3 μM carbonyl cyanide-*p*-trifluoromethoxyphenylhydrazone (FCCP), and 1 μM rotenone/antimycin A through drug injection ports. During the last injection step, cells were simultaneously stained with 2 µM Hoechst 33342 (Thermo Scientific, Rockford, IL, USA) to allow determination of cell count per well using the Cytation 5 Cell Imaging Multi-Mode Reader (Biotek, Agilent Technologies). For ISO treatment experiment, cardiomyocytes isolated from HET(52nt) and wild-type adult hearts were treated with 10 µM ISO for 24 hours prior to Seahorse assay. As the majority of zebrafish CMs (~99%) are mononucleated [9] , data were normalized to the cell number and analysed with Seahorse Wave software

**Western blotting**

Pooled zebrafish larvae were sonicated in chilled lysis buffer (150 mM NaCl, 1 mM EDTA, 0.25% sodium deoxycholate, 1% NP-40, 50 mM Tris, pH 7.4) supplemented with phosphatase inhibitors and protease inhibitor cocktail (Roche) using a sonicator (Sonopuls HD2070, Bandelin, Berlin, Germany), followed by centrifugation at 14,000 *g* for 15 minutes at 4°C. Western blotting was performed as previously described [10] . Protein extracts were resolved on 10% SDS-PAGE and transferred to PVDF membranes (Bio-Rad Laboratories, Hercules, CA, USA). The membranes were incubated with the primary antibodies followed by horseradish peroxidase-conjugated secondary antibodies. The blots were detected with Pierce™ Enhanced Chemiluminescence (ECL) substrate (Pierce Biotechnology, Rockford, lL, USA). Quantification was performed using Gel Doc Image Analyzer (Bio-Rad). The antibodies used are listed in Table S2.

**RNA extraction and real-time quantitative RT-PCR**

For detection of gene expression in zebrafish larvae or adult hearts and kidneys, RNA was extracted with miRNeasy Mini Kit (Qiagen, Hilden, Germany) according to the manufacturer's instructions. qRT-PCR was performed as described [10] . Reverse transcription was carried out using a SuperScript® VILO™ cDNA synthesis kit (Invitrogen, Carlsbad, CA, USA). Quantitative real-time PCR was performed with gene-specific primers (Table S1) and LightCycler^®^ 480 SYBR Green I Master (Roche) using Light Cycler 480 II instrument (Roche). Glyceraldehyde 3-phosphate dehydrogenase (*gapdh*), elongation factor 1 alpha 1a (*ef1*$\alpha$*1a*), and 18S ribosomal RNA (*18s rrna*) served as reference genes for normalization. RT-qPCR was performed at least three times with three technical replicates for each sample. *18s rrna* was the most stable reference gene in larvae and *gapdh* in adults.

**Histology**

Adult hearts were fixed in 10% formalin, followed by embedding in paraffin. Acid fuchsin orange G (AFOG) staining (Sigma-Aldrich, Saint Louis, MO, USA) was performed to detect fibrotic tissue according to the manufacturer's instructions. Briefly, sections were deparaffinized, post-fixed in Bouin’s solution for 60 minutes at 60^o^C, and stained with haematoxylin and AFOG solution at RT. Slides were digitally scanned using a 3DHISTECH Pannoramic 250 FLASH II (3DHISTECH Ltd., Budapest, Hungary), and quantified with HistoQuant module (3DHISTECH Ltd.). Three sections from each heart were stained and ten ventricular areas from each section were selected for quantification of the collagen- relative to myocardium- positive area with the HistoQuant program.

**Immunohistochemistry**

Paraffin sections (5 µm) from adult hearts were deparaffinized, rehydrated, and antigens retrieved by heating in citrate buffer (pH 6.0). Sections were blocked with 2% BSA in 1x PBS + 0,1% Triton X-100 and incubated with antibodies against proliferating cell nuclear antigen (Pcna) and myocyte-specific enhancer factor 2 (Mef2) (Table S2) diluted in 2% BSA in 1x PBS, followed by incubation with AlexaFluor-488 and AlexaFluor-594 conjugated secondary antibodies. Nuclei were labelled with DAPI (4′,6-Diamidino-2-Phenylindole, Dihydrochloride) (Molecular Probes, Eugene, OR, USA). Sections were mounted with ProLong Diamond antifade mountant (Molecular Probes) and imaged with a Zeiss LSM 780 confocal microscope (Carl Zeiss Microscopy GmbH, Germany). Quantitative identification of Collagen type I was performed by using EnVision™^+^ System-HRP (Dako, Carpinteria, CA, USA) according to the manufacturer's instructions. Briefly, sections were incubated with Peroxidase Block solution (Dako) and stained with anti-mouse Collagen type I antibody (DSHB, Iowa City, IA, USA) at 4 °C overnight, followed by incubation with the HRP-labelled polymer and 3,3'-diaminobenzidine (DAB) (Dako). Sections were counterstained with haematoxylin. Slides were digitally scanned using the 3DHISTECH Pannoramic 250 FLASH II (3DHISTECH Ltd.) and quantified with HistoQuant module (3DHISTECH Ltd.). Two sections from each heart were stained and the ventricular areas from each section were selected for quantification of the Collagen type I relative to myocardium-positive area with the HistoQuant program.

**Statistical analysis**

Data were analysed using Prism software (version 10.0, GraphPad, San Diego, CA, USA) and are presented as mean ± SD. One-way ANOVA with Tukey adjustment for multiple comparisons was used to calculate differences between more than two groups with normally distributed data. A two-sample unpaired two-tailed *t*-test was performed for comparisons between two groups with normally distributed data. Data that did not pass normality and lognormality tests were analysed with Mann-Whitney test to compare the difference between two groups and with Kruskal-Wallis followed by Dunn´s test in the case of three groups. A *p* value of <0.05 was assigned to be statistically significant. **Supplementary Figure 1** **CRISPR/Cas9-generated *hmox1a* mutant zebrafish.**

A. cDNA sequence of exon 3 of *hmox1a* showing the deletion of 52 bases in the mutant line KO(52del). B. Protein sequence showing a premature termination codon in the *hmox1a* mutant.

A. Danio rerio heme oxygenase 1a (*hmox1a*), mRNA

del52 1 CCACAACTTAAAACATTTTTAATTGTGTCTGCAGCTTCTGCTGTGCTCTC 50

||||||||||||||||||||||||||||||||||||||||||||||||||

exon3 1 CCACAACTTAAAACATTTTTAATTGTGTCTGCAGCTTCTGCTGTGCTCTC 50

del52 51 TATACGAGATCTACCGAGCGCTGGAGGAAGAGCTGGACAGAAACGCAGAC 100

||||||||||||||||||||||||||||||||||||||||||||||||||

exon3 51 TATACGAGATCTACCGAGCGCTGGAGGAAGAGCTGGACAGAAACGCAGAC 100

del52 101 CACCCAGCAGTGCAGCCCATTTACTTCCCTCAGGAATTGGCCAGA----- 145

|||||||||||||||||||||||||||||||||||||||||||||

exon3 101 CACCCAGCAGTGCAGCCCATTTACTTCCCTCAGGAATTGGCCAGACTGGA 150

del52 146 -----------------------------------------------AGA 148

|||

exon3 151 GGCTCTGGGGCAGGACTTGGAGCACTTCTTCGGCCCCCAGTGGAGGAAGA 200

del52 149 GAATAACAGTGCCTGCCGCCACACACCGATATGCACAGAGACTGAGAGAG 198

||||||||||||||||||||||||||||||||||||||||||||||||||

exon3 201 GAATAACAGTGCCTGCCGCCACACACCGATATGCACAGAGACTGAGAGAG 250

del52 199 GTGACTGTGTGAACCTGCTCTTTTTAGGATATGAAACTGCATCGCTTTTA 248

||||||||||||||||||||||||||||||||||||||||||||||||||

exon3 251 GTGACTGTGTGAACCTGCTCTTTTTAGGATATGAAACTGCATCGCTTTTA 300

B. CLUSTAL 2.1 multiple sequence alignment

Hmox1a_del52 LLLCSLYEIYRALEEELDRNADHPAVQPIYFPQELARRE*stop

Hmox1a_wt LLLCSLYEIYRALEEELDRNADHPAVQPIYFPQELARLEALGQDLEHFFG

**************************************

**Supplementary Figure 2** **RT-qPCR analyses of the genes involved in mitochondrial OXPHOS and erythropoiesis.**

A-B RT-qPCR analyses showing no significant alteration of complex IV subunit *mt-co1* (A) and complex I subunit *mt-nd1* (B) in KO(52del) hearts compared to WT controls. *mt-co1, cytochrome c oxidase subunit 1*; *mt-nd1, NADH dehydrogenase subunit 1*. The graphs represent the quantification of two individual analyses of RNA extracts from pooled samples of two-three hearts. Each analysis includes three replicates. WT *n* = 19; HET(52del) *n* = 19; KO(52del) *n* =19.

C Mitochondria DNA abundance in adult zebrafish hearts. Results are expressed as mitochondrial DNA amount relative to genomic DNA. The graphs represent the quantification of two individual analyses of DNA extracts from pooled samples of three-four hearts. Each analysis includes three replicates. WT *n* = 10; HET(52del) *n* = 12; KO(52del) *n* = 10.

D-F The expression of erythropoietin gene *epoa* (D), erythroid transcription regulators, *klfd* (E) and *gata1a* (F), did not differ significantly among the three genotypic groups. *epoa, erythropoietin a*; *klfd, krüppel-like factor d*; *gata1a*, *GATA binding protein 1a*. The graphs represent the quantification of two individual analyses of RNA extracts from pooling samples of two kidneys. Each analysis includes three replicates. WT *n* = 10; HET(52del) *n* = 10; KO(52del) *n* = 10.

**
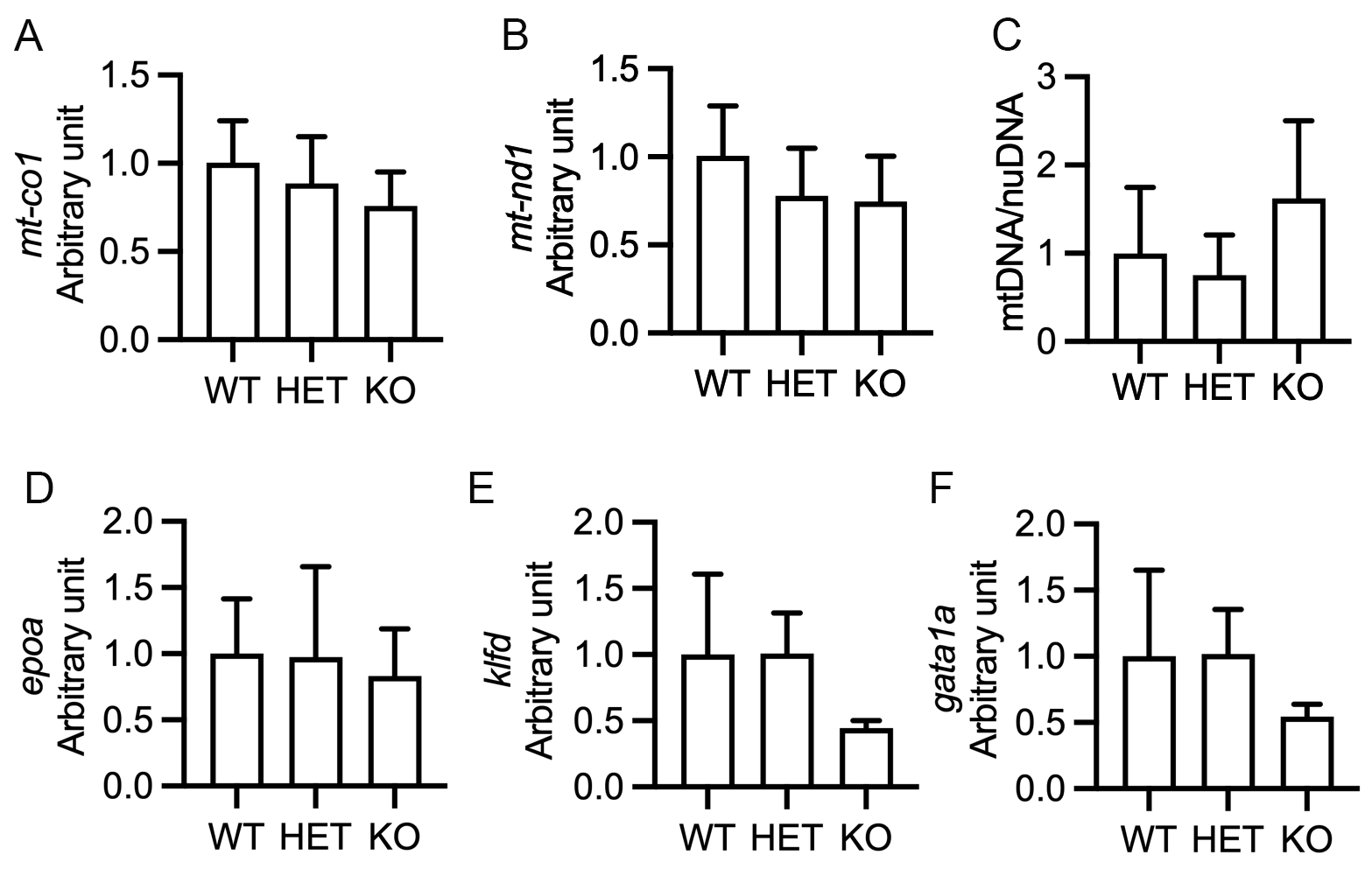
**Data are presented as mean ± SD. One-way ANOVA with Tukey adjustment for multiple comparisons.

**Supplementary Figure 3** **Loss of *hmox1a* has no significant impact on cardiac response to ISO in larvae.**

Embryos at 2 dpf received ISO for 4 days and cardiac function was monitored at 6 dpf. A-E, ISO boosts cardiac output (A), stroke volume (B), heart rate (C), ejection fraction (D), and end-diastolic area (E) in all three genotypic groups. F, RT-qPCR analyses of the expression of *hmox1a* in ISO-induced larvae compared to vehicle-treated controls.

WT, vehicle *n* = 10, ISO *n* = 10; HET, vehicle *n* = 9, ISO *n* = 9; KO, vehicle *n* = 10, ISO *n* = 10. Data are presented as mean ± SD. Two-sample *t*-test. * *p* < 0.05, ** *p* < 0.01, *** *p* < 0.001.


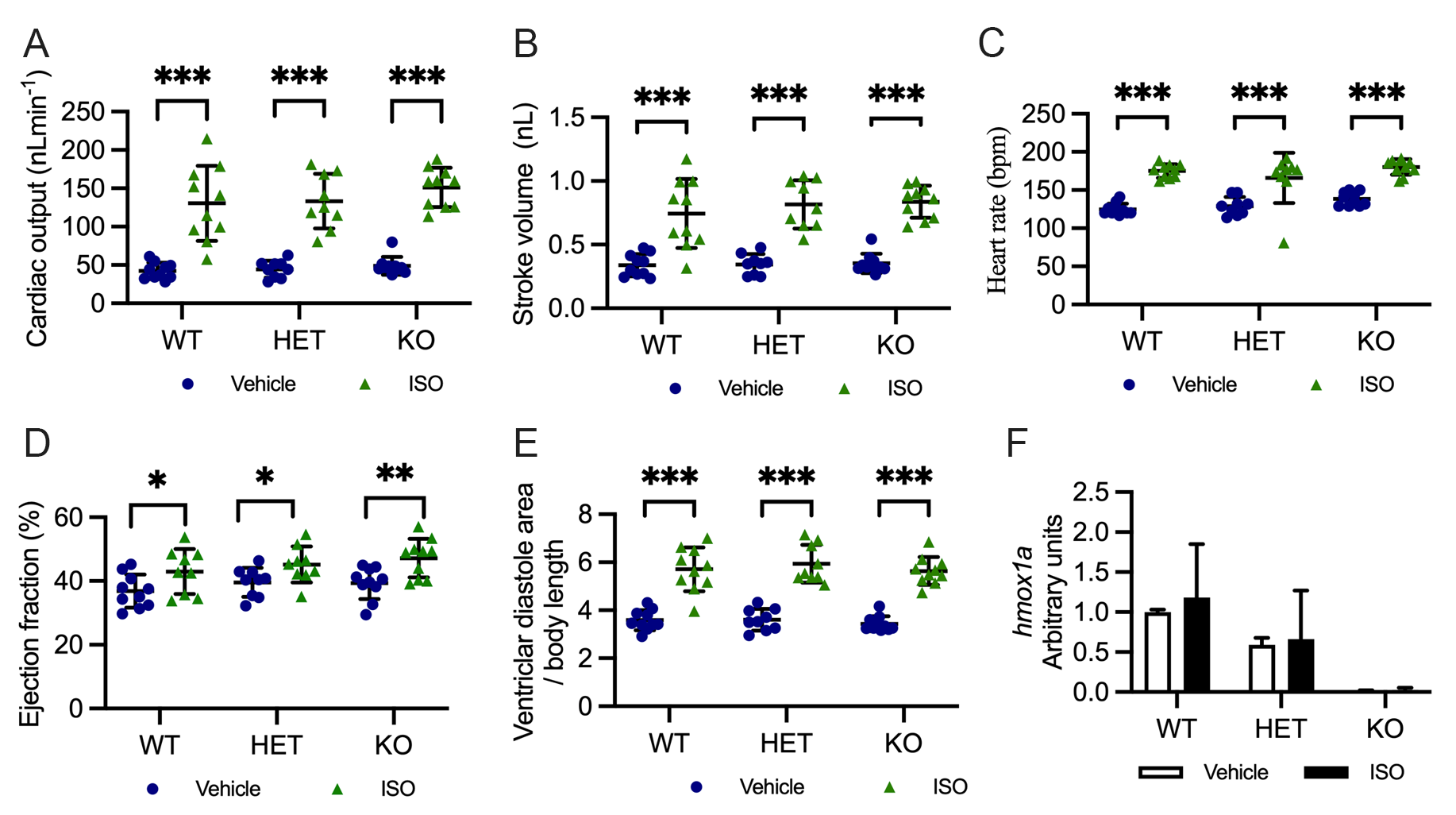


**Supplementary Figure 4 Cardiac responses to different ISO dosage in wild-type adult zebrafish.**

ISO treatment at a dosage of 150 mg/kg results in reduced cardiac output (A) and a tendency of reduced stroke volume (*p*=0.07) (B) without significant effect on heart rate (C) or end-diastolic (E) and end-systolic area (F) compared to vehicle. ISO treatment at a dosage of 75 mg/kg has no significant effect on cardiac function (A-F). Ejection fraction (D) in zebrafish treated with 150 mg/kg ISO appears lower than that in zebrafish treated with 75 mg/kg.

Vehicle *n* = 14, ISO 75 mg/kg *n* = 10, ISO 150 mg/kg *n* = 5.

Data are presented as mean ± SD. One-way ANOVA with Tukey adjustment for multiple comparisons. * *p* < 0.05.


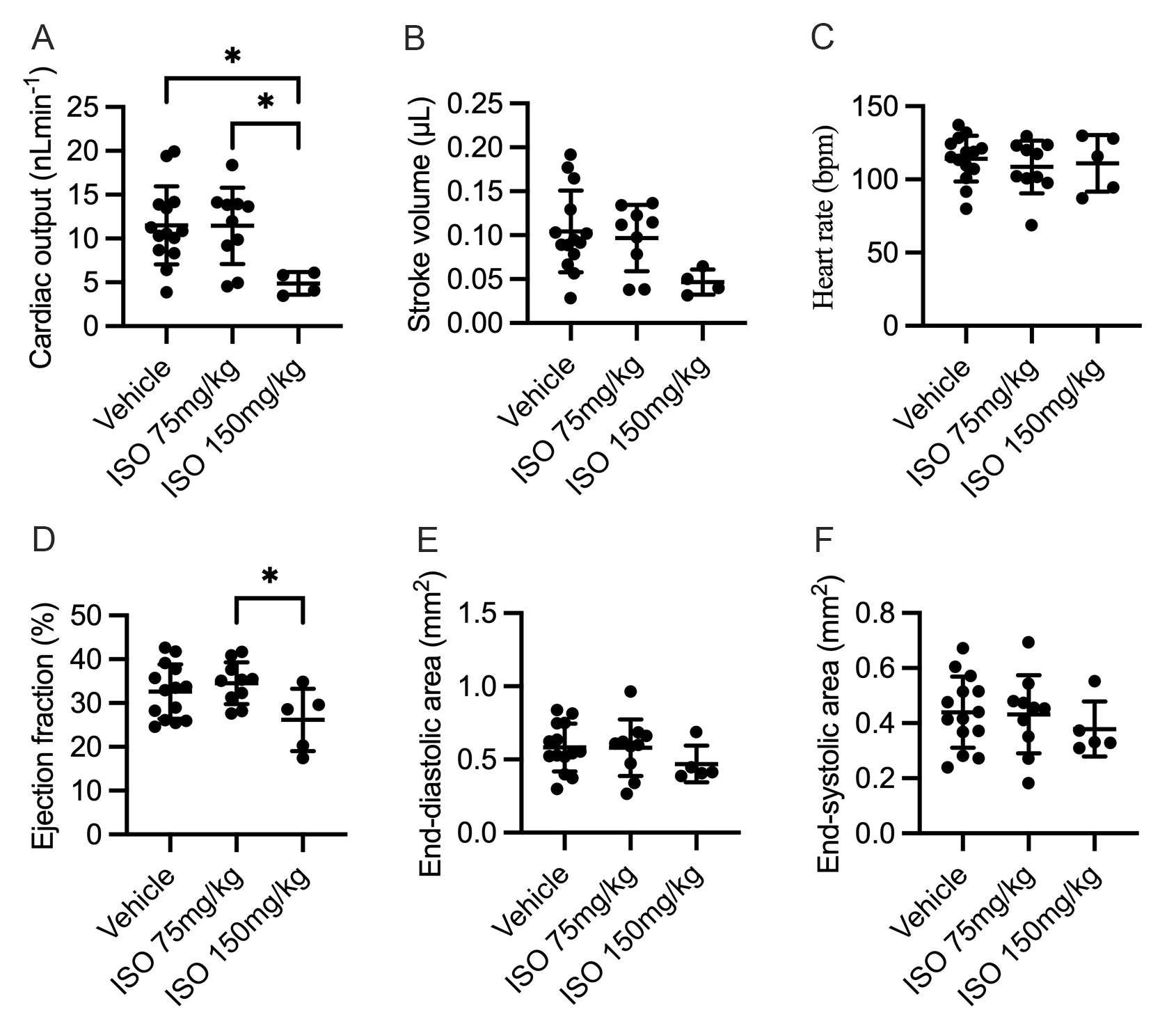


**Supplementary Figure 5** **Original immunoblots of β-tubulin (A) and Gapdh (B) in WT, HET(52del), and KO(52del) larvae under normoxic or hypoxic (3% O_2_ for 24 h) condition.** Norm. normoxia; Hypox. Hypoxia.


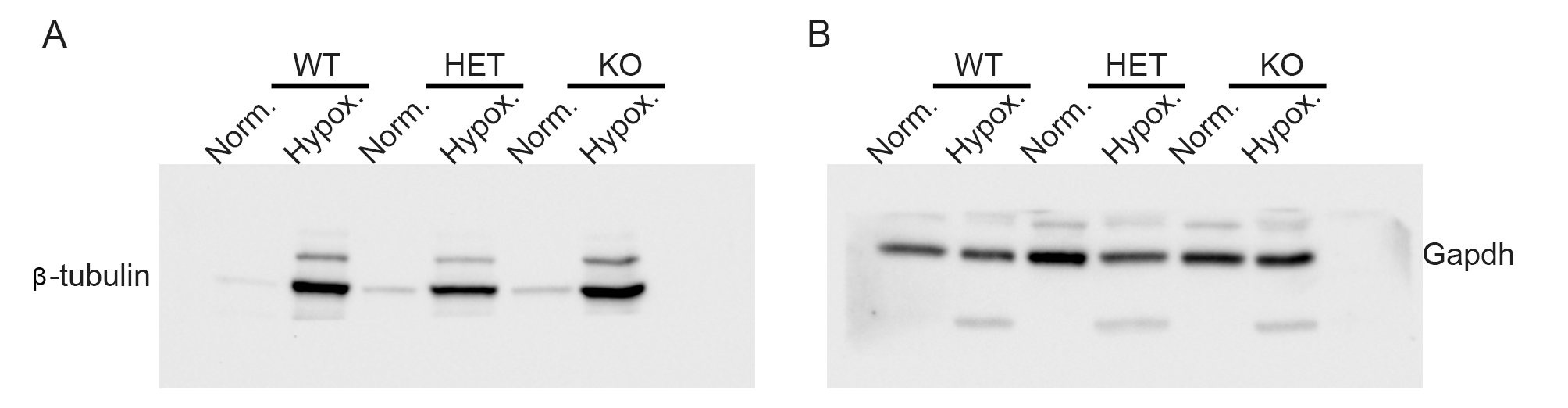


**Supplementary Table 1. List of gRNA and primers used in the study.**

| Target | Assay | Primer sequences | Reference |
| --- | --- | --- | --- |
| *hmox1a* | HRM | Forward 5´- GGGGTCACTTGATTGCTTTTGAG -3´  Reverse 5´- CGCTCGTCACTCCAGGAAAC -3´ | This study |
| *hmox1a* | RT-qPCR | Forward 5´- TTGGCCAGACTGGAGGCTCT -3´  Reverse 5´- CGAGGTAGCGGGTGTAAGCG -3´ | This study |
| *hmox1b* | RT-qPCR | Forward 5´- GCAGTGATCTGTCTGAACAG -3´  Reverse 5´- GCTTGTACTGTGTTTGTGTG -3´ | [11] |
| *hmox2a* | RT-qPCR | Forward 5´- ATGGCGGTCAGTGGAAACACAACC -3  Reverse 5´- GGCAACAGCAGCAACCAATGTGGC -3´ | [11] |
| *hmox2b* | RT-qPCR | Forward 5´- TTTAGGAGGTTGAGTTGGAGTCAG -3´  Reverse 5´- TTCTGCCTTCTGGTGCACTTCT -3´ | [11] |
| *mt-nd1* | RT-qPCR | Forward 5´- CCCACGATTCCGATACGACC -3´  Reverse 5´- GTGCGATTGGTAGGGCGATA -3´ | This study |
| *sdhb* | RT-qPCR | Forward 5′- GCTGGATGATCGACTCTCGG -3′  Reverse 5′- TTCCTGGATTCAGCCCCTTG -3′ | This study |
| *mt-co1* | RT-qPCR | Forward 5′- TGAGAAGCCTTTACCGCCAA -3′  Reverse 5′- ACGAATGCTGGCTCCTCAAA -3′ | This study |
| *nuDNA* | RT-qPCR | Forward 5′- ATGGGCTGGGCGATAAAATTGG -3′  Reverse 5′- ACATGTGCATGTCGCTCCCAAA -3′ | [12] |
| *16s rRNA* | RT-qPCR | Forward 5′- CAAACACAAGCCTCGCCTGTTTAC -3′  Reverse 5′- CACTGACTTGATGGGGGAGACAGT -3′ | This study |
| *txnrd3* | RT-qPCR | Forward 5′- GGCCTCGTTACCTGGGCATC -3′  Reverse 5′- ACCAGCCAGAAAGCCACCAC -3′ | This study |
| *sod2* | RT-qPCR | Forward 5′- GTGCGCTGCAACCTTCAACC -3′  Reverse 5′- TGTGAGGTCAGGGAGAGCGT -3′ | This study |
| *foxo3a* | RT-qPCR | Forward 5′- TAGCAGCATGCAGCGACTCC -3′  Reverse 5′- CGAGCTCTCGATGGCTTGGG -3′ | This study |
| *sirt3* | RT-qPCR | Forward 5′- ACCAACCCAACCTGACGCAC -3′  Reverse 5′- AATGCCAGCCATGCGCTCAA -3′ | This study |
| *gata1a* | RT-qPCR | Forward 5′- CAGTTCAGCAGCGCTCTATTCA -3′  Reverse 5′- AGCCTCAGGTGGCGAAAGT -3′ | [13] |
| *klfd* | RT-qPCR | Forward 5′- TGTCACCTGAACGGTCCAACT -3′  Reverse 5′- GGTGCGTTCAGCTCCTGATAA -3′ | [13] |
| *epoa* | RT-qPCR | Forward 5′- GCCTGTCCTCCCCATTACGC -3′  Reverse 5′- CCTCTGCATCCCATGCCTCC -3′ | This study |
| *epor* | RT-qPCR | Forward 5′- CACATGGGCAAGGTGGAGGA -3′  Reverse 5′- GTCCGGTTTGACACGGACCC -3′ | This study |
| *hbαa1* | RT-qPCR | Forward 5′- CTGAAGCCCTCGCCAGAATGC -3′  Reverse 5′- TCTTCACGGGACCAGACCCA -3′ | This study |
| *hbβa1* | RT-qPCR | Forward 5′- CCCGACAACTTCAGGCTTCTC -3′  Reverse 5′- AGAAACTTCTGCCAGGCCTCC -3′ | This study |
| *gapdh* | RT-qPCR | Forward 5′- CAGGCATAATGGTTAAAGTTGGTA -3′  Reverse 5′- CATGTAATCAAGGTCAATGAATGG -3′ | [14] |
| *18S rRNA* | RT-qPCR | Forward 5´- GCCTGCGGCTTAATTTGACT -3´  Reverse 5´- ACCACCCACAGAATCGAGAAA -3´ | This study |
| *ef1α1a* | RT-qPCR | Forward 5´- GAGACGCGGCCATTGTGGAA -3´  Reverse 5´- ACCGTCTGACGCATGTCACG -3´ | This study |

**Supplementary Table 2. List of antibodies used in the study.**

| Antibody | Host | Manufacturer | Reference |
| --- | --- | --- | --- |
| β-Tubulin | Mouse monoclonal | Millipore | 05-661 |
| GAPDH | Rabbit polyclonal | Abcam^®^ | Ab9485 |
| PCNA | Mouse monoclonal | Cell signaling | 2586 |
| MEF-2 | Rabbit polyclonal | Santa Cruz | Sc-313 |
| COL1A1 | Mouse monoclonal | DSHB | SP1.D8 |
| HRP-rabbit IgG | Goat polyclonal | Jackson ImmunoResearch | 111-035-003 |
| HRP-mouse IgG | Goat polyclonal | Jackson ImmunoResearch | 115-035-044 |
| Alexa Fluor® 488-mouse IgG | Goat polyclonal | Invitrogen | A-11001 |
| Alexa Fluor® 488-rabbit IgG | Goat polyclonal | Invitrogen | A-11008 |
| AlexaFluor® 594-rabbit IgG | Goat polyclonal | Invitrogen | A-11012 |
| AlexaFluor® 546-goat IgG | Donkey polyclonal | Invitrogen | A-11056 |

**References**

[1] **Hwang WY, Fu Y, Reyon D, et al.** Efficient genome editing in zebrafish using a CRISPR-Cas system. *Nat.Biotechnol.* 2013; 31; 227–9.

[2] **Varshney GK, Carrington B, Pei W, et al.** A high-throughput functional genomics workflow based on CRISPR/Cas9-mediated targeted mutagenesis in zebrafish. *Nat.Protoc.* 2016; 11; 2357–75.

[3] **Chen YC, Baronio D, Semenova S, et al.** Cerebral Dopamine Neurotrophic Factor Regulates Multiple Neuronal Subtypes and Behavior. *J.Neurosci.* 2020; 40; 6146–64.

[4] **Dash SN, Narumanchi S, Paavola J, et al.** Sept7b is required for the subcellular organization of cardiomyocytes and cardiac function in zebrafish. *Am.J.Physiol.Heart Circ.Physiol.* 2017; 312; H1085–95.

[5] **Wang LW, Huttner IG, Santiago CF, et al.** Standardized echocardiographic assessment of cardiac function in normal adult zebrafish and heart disease models. *Dis Model Mech* 2017; 10; 63–76.

[6] **Narumanchi S, Kalervo K, Perttunen S, et al.** Inhibition of let-7c Regulates Cardiac Regeneration after Cryoinjury in Adult Zebrafish. *J Cardiovasc Dev Dis* 2019; 6; E16.

[7] **Grimm D, Elsner D, Schunkert H, et al.** Development of heart failure following isoproterenol administration in the rat: role of the renin-angiotensin system. *Cardiovasc Res* 1998; 37; 91–100.

[8] **Sander V, Suñe G, Jopling C, et al.** Isolation and in vitro culture of primary cardiomyocytes from adult zebrafish hearts. *Nat Protoc* 2013; 8; 800–9.

[9] **González-Rosa JM, Sharpe M, Field D, et al.** Myocardial Polyploidization Creates a Barrier to Heart Regeneration in Zebrafish. *Developmental Cell* 2018; 44; 433-446.e7.

[10] **Wang H, Segersvärd H, Siren J, et al.** Tankyrase Inhibition Attenuates Cardiac Dilatation and Dysfunction in Ischemic Heart Failure. *Int J Mol Sci* 2022; 23; 10059.

[11] **Holowiecki A, O’Shields B, Jenny MJ**. Characterization of heme oxygenase and biliverdin reductase gene expression in zebrafish (Danio rerio): Basal expression and response to pro-oxidant exposures. *Toxicol.Appl.Pharmacol.* 2016; 311; 74–87.

[12] **Hunter SE, Jung D, Di Giulio RT, et al.** The QPCR assay for analysis of mitochondrial DNA damage, repair, and relative copy number. *Methods* 2010; 51; 444–51.

[13] **Kulkeaw K, Ishitani T, Kanemaru T, et al.** Cold exposure down-regulates zebrafish hematopoiesis. *Biochem Biophys Res Commun* 2010; 394; 859–64.

[14] **Burkhalter MD, Sridhar A, Sampaio P, et al.** Imbalanced mitochondrial function provokes heterotaxy via aberrant ciliogenesis. *J.Clin.Invest.*; 129; 2841–55.
